# Hydrogen–Deuterium Exchange Mass Spectrometry Reveals Mechanistic Insights into RNA Oligonucleotide-Mediated Inhibition of TDP-43 Aggregation

**DOI:** 10.1021/jacs.4c11229

**Published:** 2024-11-29

**Authors:** Thomas C. Minshull, Emily J. Byrd, Monika Olejnik, Antonio N. Calabrese

**Affiliations:** Astbury Centre for Structural Molecular Biology, School of Molecular and Cellular Biology, Faculty of Biological Sciences, University of Leeds, Leeds LS2 9JT, U.K.

## Abstract

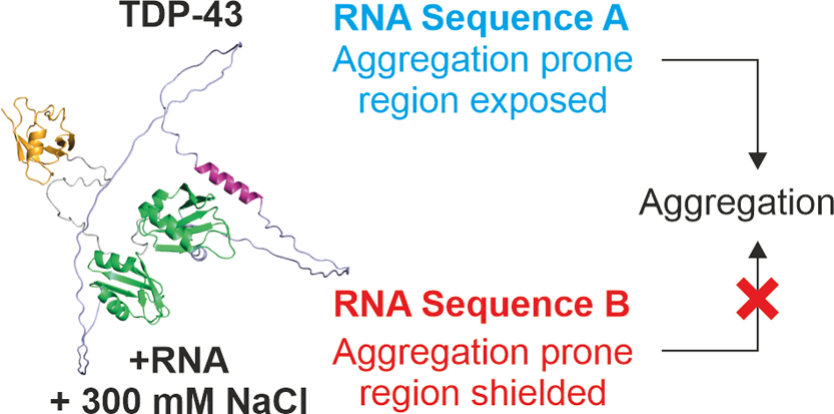

Deposits of aggregated TAR DNA-binding protein 43 (TDP-43) in the brain are associated with several neurodegenerative diseases. It is well established that binding of RNA/DNA to TDP-43 can prevent TDP-43 aggregation, but an understanding of the structure(s) and conformational dynamics of TDP-43, and TDP-43-RNA complexes, is lacking, including knowledge of how the solution environment modulates these properties. Here, we address this challenge using hydrogen–deuterium exchange-mass spectrometry. In the presence of RNA olignoucleotides, we observe protection from exchange in the RNA recognition motif (RRM) domains of TDP-43 and the linker region between the RRM domains, consistent with nucleic acid binding modulating interdomain interactions. Intriguingly, at elevated salt concentrations, the extent of protection from exchange is reduced in the RRM domains when bound to an RNA sequence derived from the 3′ UTR of the TDP-43 mRNA (CLIP34NT) compared to when bound to a (UG)6 repeat sequence. Under these conditions, CLIP34NT is no longer able to prevent TDP-43 aggregation. This suggests that a salt-induced structural rearrangement occurs when bound to this RNA, which may play a role in facilitating aggregation. Additionally, upon RNA binding, we identify differences in exchange within the short α-helical region located in the C-terminal domain (CTD) of TDP-43. These allosterically altered regions may influence the ability of TDP-43 to aggregate and fine-tune its RNA binding repertoire. Combined, these data provide additional insights into the intricate interplay between TDP-43 aggregation and RNA binding, an understanding of which is crucial for unraveling the molecular mechanisms underlying TDP-43-associated neurodegeneration.

## Introduction

Frontotemporal lobar degeneration (FTLD) and amyotrophic lateral sclerosis (ALS) are related neurodegenerative disorders that share a common pathology associated with cytoplasmic proteinaceous deposits in degenerating neurons.^1−4^ These insoluble deposits comprise the DNA/RNA binding protein TAR DNA binding protein 43 (TDP-43) in 97 and 45% of ALS and FTLD cases, respectively.^4^ Cytoplasmic TDP-43 inclusions are also found in patients with other neurodegenerative diseases, e.g., Alzheimer’s and Parkinson’s.^5^ Under normal cellular conditions, TDP-43 is located within the nucleus, but its function, especially in cellular stress responses, relies on controlled shuttling between the nucleus and cytoplasm.^6,7^ Cytoplasmic TDP-43 aggregates found in neurons and glial cells of patients are known to contain ubiquitinated, truncated (predominantly comprising the disordered C-terminal domain [CTD] in isolation) and phosphorylated TDP-43.^8−10^ Structures of fibrils isolated from brain tissue of individuals with ALS/FTLD have been solved using cryo-electron microscopy (EM), and demonstrated that TDP-43 fibrils from patients adopt distinct architectures in different disease pathologies.^11−13^ However, the mechanisms by which aggregation pathways are fine-tuned in different disease states, resulting in different fibril polymorphs, remain undetermined for all amyloidogenic proteins, including TDP-43.^14,15^ TDP-43, and truncations comprising the disordered CTD, have also been shown to undergo liquid–liquid phase separation (LLPS) *in vitro* and in cell.^16^

TDP-43 plays a key role in RNA metabolism, including in transcription, RNA splicing, and RNA transport.^17,18^ This diverse repertoire of functions is consistent with evidence demonstrating that TDP-43 is able to bind an array of RNA targets (>6000 pre-mRNA targets of TDP-43 have been identified within the brain).^19−23^ TDP-43 favors binding to UG repeat motifs, such as those found in intronic regions,^19^ which highlights its essential role within alternative splicing. Due to its critical involvement in RNA metabolism, and its tendency to aggregate, cellular levels of TDP-43 need to be tightly controlled.^24,25^ As a consequence, autoregulation of TDP-43 levels is controlled via a negative feedback loop whereby TDP-43 binds to an approximately 500 nucleotide region in the 3′ untranslated region (UTR) of its own mRNA transcript (TARDBP), to impair translation.^26^ Cross-linking immunoprecipitation (CLIP) has identified a short 34-nucleotide segment in the 3′ UTR of TARDP (called CLIP34NT) to which TDP-43 binds with high affinity,^19,20,26^ and data has shown that this binding is protective from aggregation in neuronal cell models of TDP-43 proteinopathies^27^ and modulates TDP-43 LLPS.^28,29^ Additional evidence from *in vitro*(30) and in cell^27^ studies suggest a role for RNA in modulating TDP-43 aggregation, amyloid assembly and disease pathology. However, the mechanistic basis of how different RNA sequences afford differential effects on amyloid assembly, and how this relates to disease pathogenesis and amyloid fibril morphology remains unknown.

**Figure 1:**
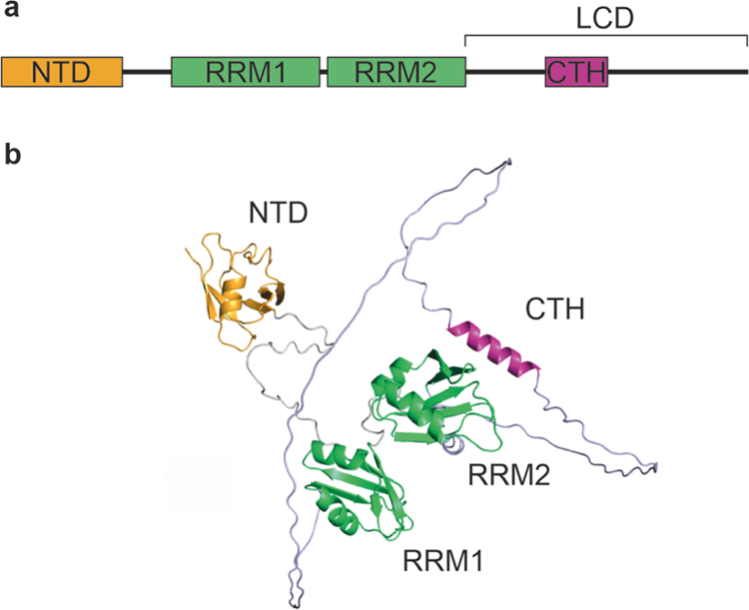
Architecture of TDP-43. (a) Domain architecture of TDP-43. Folded regions/domains are colored orange (N-terminal domain, NTD), green (RNA recognition motifs RRM1 and RRM2), and purple (C-terminal helix, CTH). The low complexity domain (LCD) is also indicated. (b) AlphaFold 2 model of TDP-43. Regions are colored as in (a), linker regions between folded domains are shown in gray and the LCD is colored in pale blue.

Structurally, TDP-43 is a 414 amino acid protein comprising three structured domains: the N-terminal domain (NTD), and two RNA recognition motifs (RRM1 and RRM2)^31−33^ (Figure 1a,b). Among these domains are unstructured linker regions and the disordered C-terminal domain (CTD), often referred to as the low complexity domain (LCD), owing to its low amino acid variation.^34^ Within the LCD there is a small α helix (here called the C-terminal helix, CTH, Figure 1a,b) that has been shown to regulate the ability of TDP-43 to undergo LLPS.^35−37^ There is growing evidence for the importance of crosstalk between the domains within TDP-43 for its function. For example, mutations in and around the CTH influence not only the ability of the CTD of TDP-43 to undergo LLPS within the cell,^35,36^ but also the RNA sequence binding preference of TDP-43.^38^ As a result, understanding how the different domains of TDP-43 are involved in its RNA binding function is crucial in elucidating TDP-43’s mechanism of action in RNA metabolism, as well as its role in disease pathology, and is potentially vital for developing new therapeutics.^39^ In the context of disease, it is well established that RNA can inhibit TDP-43 aggregation,^40,41^ but the molecular basis of this protective function is not well understood, particularly in the context of full length TDP-43.

In this study, we aimed to elucidate how full length TDP-43 binds to two model RNA oligonucleotide targets: a 12mer oligonucleotide UG(6) and an oligonucleotide derived from the 3′ UTR of the TDP-43 mRNA sequence: CLIP34NT.^22,26,32^ We report key differences between UG(6) and CLIP34NT in protecting TDP-43 from aggregation, especially under conditions whereby NaCl concentrations are elevated. Electrostatic interprotein interactions are important in tuning the conformational landscape of disordered proteins,^42^ and salt and buffer conditions tune LLPS and aggregation,^43,44^ but it is difficult to elucidate how changes in the solution environment alter the dynamics of disordered proteins and their interactions. We observed that at increased NaCl concentrations (300 mM), CLIP34NT is no longer able to antagonize TDP-43 aggregation as efficiently, even when high levels of protein are bound to RNA, highlighting the importance of both solution conditions and RNA oligonucleotide sequence in fine-tuning TDP-43 aggregation. Therefore, to understand the structural basis of these observations, we utilized hydrogen–deuterium exchange mass spectrometry (HDX-MS) to probe for structural differences upon TDP-43 binding to the two different RNA sequences we have investigated and found an increased degree of protection from deuterium uptake at some, but not all, RNA binding motifs within the RRM domains in the presence of UG(6) compared to CLIP34NT. This provides new insight into the cooperative mechanism of RNA binding to TDP-43 and highlights how different RNA sequences and structures within mRNA may be selected and bound by the RNA-binding motifs/domains in TDP-43. Interestingly, we identified allosteric impacts of RNA binding on TDP-43 in the CTH, which is crucial for TDP-43 LLPS and its ability to undergo many protein–protein interactions,^35,38,45,46^ where we observe deprotection from deuterium exchange upon RNA binding. Moreover, we observe that when TDP-43 is bound to CLIP34NT, under conditions where aggregation was not prevented (300 mM NaCl), an extensive reduction in the levels of hydrogen exchange in the RRM domains was apparent compared to when TDP-43 was bound to UG(6) (which is able to prevent TDP-43 aggregation), suggesting a structural basis for how sequence specific RNA binding to the RRM domains modulates TDP-43 aggregation.^30,47^ Taken together, this study reveals new insights into the structure and dynamics of TDP-43, alongside the intraprotein and protein-RNA interactions which likely play a key role in fine-tuning TDP-43 self-assembly into higher order aggregates and ultimately amyloid fibrils. Additionally, this work highlights the power of an integrative structural proteomics approach to interrogate the structure and dynamics of intrinsically disordered proteins and their interactions with nucleic acids, along with how solution conditions tune these properties.

## Methods

### Protein Expression

Plasmid pJ4M/TDP-43 was a gift from Nicolas Fawzi (Addgene plasmid # 104480; http://n2t.net/addgene:104480; RRID:Addgene_104480).^48^ The vector was transformed into BL21 (DE3) cells, and expression and purification of TDP-43 with a C-terminal maltose binding protein (MBP) tag (TDP-43-MBP) was carried out based on a previously described method.^48^ Briefly, cell cultures were grown in LB medium containing 50 μg/mL kanamycin at 37 °C with shaking (200 rpm) until the culture reached an OD_600_ of ∼0.6. The temperature was then lowered to 16 °C, after which protein expression was induced by the addition of 1 mM IPTG. Following overnight incubation at 16 °C with shaking (200 rpm), cells were harvested by centrifugation and resuspended in TDP-43 binding buffer (20 mM Tris–Cl pH 8.0, 1 M NaCl, 10 mM imidazole, 10% (v/v) glycerol, 1 mM DTT) supplemented with cOmplete EDTA-free protease inhibitor cocktail (Roche). Cells were then lysed using a cell disruptor (Constant Cell Disruption Systems). The cell lysate was incubated with DNaseI under constant agitation at room temperature for 20 min, and then clarified by centrifugation, applied to a 5 mL HisTrap HP column (Cytiva), and washed with five volumes of TDP-43 binding buffer. TDP-43-MBP was eluted with a linear gradient of TDP-43 binding buffer to TDP-43 elution buffer (20 mM Tris–Cl pH 8.0, 1 M NaCl, 500 mM imidazole, 10% (v/v) glycerol, 1 mM DTT) over 20 column volumes. Fractions corresponding to TDP-43-MBP were pooled and concentrated using a 30 kDa MWCO centrifugal ultrafiltration device (Vivaspin, Sartorius) to ∼5 mL. The protein was then further purified by size exclusion chromatography using a Superdex 200 26/60 column (Cytiva) equilibrated with 20 mM Tris–Cl pH 8.0, 300 mM NaCl, 1 mM DTT. Purified TDP-43-MBP was concentrated to ∼120 μM using a 30 kDa MWCO centrifugal ultrafiltration device (Vivaspin, Sartorius), flash frozen, and stored at −80 °C.

### Microscale Thermophoresis

MST experiments were conducted on a Monolith NT.115 system (NanoTemper Technologies). RNA oligonucleotides were purchased 5′ labeled with fluorescein (FAM) (Eurofins). TDP-43-MBP was buffer exchanged (Zeba Spin Desalting Columns, ThermoFisher Scientific) into either 20 mM HEPES pH 7.4, 150 mM NaCl or 50 mM potassium phosphate pH 8.0, 300 mM NaCl, and the protein was diluted to a concentration of 90 μM. This stock solution was used to create a serial dilution series in the appropriate buffer. A solution of FAM-labeled RNA oligonucleotides was added to the protein 1:1 (v/v) to give a final RNA concentration of 79 nM, and the protein concentrations were 30 000–0.92 nM for experiments involving CLIP34NT and UG(17), or 2095–28 nM for experiments involving UG(6). The samples were loaded into premium-coated capillaries (NanoTemper Technologies) and MST experiments were conducted in duplicate. Data were fitted using a variable slope agonist vs response model implemented in GraphPad Prism 9.4.1 (GraphPad Software) to determine EC_50_ values and Hill coefficients (Supporting Table 1).

### Nephelometry

TDP-43-MBP was buffer exchanged into the appropriate buffer (20 mM HEPES, 150 mM NaCl pH 7.4 or 50 mM potassium phosphate, 300 mM NaCl, pH 8.0) immediately prior to analysis (Zeba Spin Desalting Columns, ThermoFisher Scientific) and diluted to a concentration of 10 μM. Aggregation was initiated by addition of Tobacco Etch Virus (TEV) protease to the solutions (1:20 TEV/protein molar ratio). Light scattering of 50 μL of each solution in a 96-well plate (Corning Product No. 3881) was then monitored using a Nephelostar (BMG Labtech GmbH) using an excitation wavelength of 635 ± 10 nm, over 6 h at 25 °C. RNA oligonucleotide concentrations were added to give 49 and 89% bound (Supporting Table 2). The signal of a buffer blank was subtracted, and the starting value in each data set was set as zero.

### Hydrogen–Deuterium Exchange Mass Spectrometry

For HDX-MS experiments, a robot for automated HDX (LEAP Technologies) was coupled to a Acquity M-Class LC and HDX manager (Waters). Samples comprised protein (TDP-43-MBP), with or without RNA [UG(6) or CLIP34NT]. Samples were prepared in either 50 mM potassium phosphate pH 8, 300 mM NaCl or 20 mM HEPES pH 7.4, 150 mM NaCl. In all experiments, TDP-43-MBP was at a concentration of 10 μM and the RNA concentration was changed depending on the experiment to achieve 49% bound (to enable direct comparison between all RNA-bound states). These RNA concentrations were chosen due to the occurrence of RNA induced signal suppression at higher concentrations, as others have shown.^49^ Despite not achieving complete protein saturation with RNA, peptides with EX1 or EXX deuterium uptake kinetics are not an obvious feature of our data. In 20 mM HEPES pH 7.4, 150 mM NaCl, RNA concentrations were 11.8 μM for UG(6) or 12.4 μM for CLIP34NT. In 50 mM potassium phosphate pH 8, 300 mM NaCl, RNA concentrations were 12 μM for UG(6) or 28 μM for CLIP34NT. To initiate the HDX experiment, 95 μL of deuterated buffer (50 mM potassium phosphate pD 8.0, 300 mM NaCl or 20 mM HEPES pD 7.4, 150 mM NaCl) was added to 5 μL of protein-containing solution, and the mixture was incubated at 4 °C for 0.5, 2, or 5 min. For each time point and condition, three replicate measurements were performed. The HDX reaction was quenched by adding 100 μL of quench buffer (10 mM potassium phosphate, 0.05% DDM, pH 2.2) to 50 μL of the labeling reaction. To generate the fully deuterated sample, TDP-43 was buffer exchanged into MS-grade water, then 20 μL of 10 μM protein was placed into a low protein-binding tube, and vacuum concentrated to dryness. Once dry, samples were resuspended in the respective deuterated buffers supplemented with 8 M *d*_4_-urea and incubated for 24 h at 4 °C. Samples were then quenched [by adding 100 μL of quench buffer (10 mM potassium phosphate, 0.05% DDM, pH 2.2) to 50 μL of the labeling reaction] and analyzed as detailed below.

The quenched sample (50 μL) was proteolyzed by flowing through an immobilized pepsin column (Enzymate, Waters). The produced peptides were trapped on a VanGuard Precolumn [Acquity UPLC BEH C18 (1.7 μm, 2.1 mm × 5 mm, Waters)] for 3 min and the peptides were separated using a C18 column (75 μm × 150 mm, Waters, UK) by gradient elution of 0–40% (v/v) acetonitrile (0.1% v/v formic acid) in H_2_O (0.3% v/v formic acid) over 7 min at 40 μL min^–1^.

Peptides were detected using a Synapt G2Si mass spectrometer (Waters) operating in HDMS^E^ mode, with dynamic range extension enabled. IM separation was used to separate peptides prior to CID fragmentation in the transfer cell. CID data were used for peptide identification, and uptake quantification was performed at the peptide level. Data were analyzed using PLGS (v3.0.2) and DynamX (v3.0.0) software (Waters). Search parameters in PLGS were as follows: peptide and fragment tolerances = automatic, min fragment ion matches = 1, digest reagent = nonspecific, false disco rate = 4. Restrictions for peptides in DynamX were as follows: minimum intensity = 1000, minimum products per amino acid = 0.3, max sequence length = 25, max ppm error = 5, file threshold = 3. The software Deuteros 2.0 was used to identify peptides with statistically significant increases/decreases in deuterium uptake and to prepare Wood’s plots.^50^ The raw HDX-MS data have been deposited to the ProteomeXchange Consortium via the PRIDE^51^ partner repository with the data set identifier PXD054930. A summary of the HDX-MS data, as recommended by reported guidelines is shown in Supporting Table 3. Sequence coverage maps of TDP-43 are shown in Supporting Figures 1 and 2.

## Results

### HDX-MS Reveals the Effect of NaCl on the Conformational Dynamics of TDP-43

**Figure 2:**
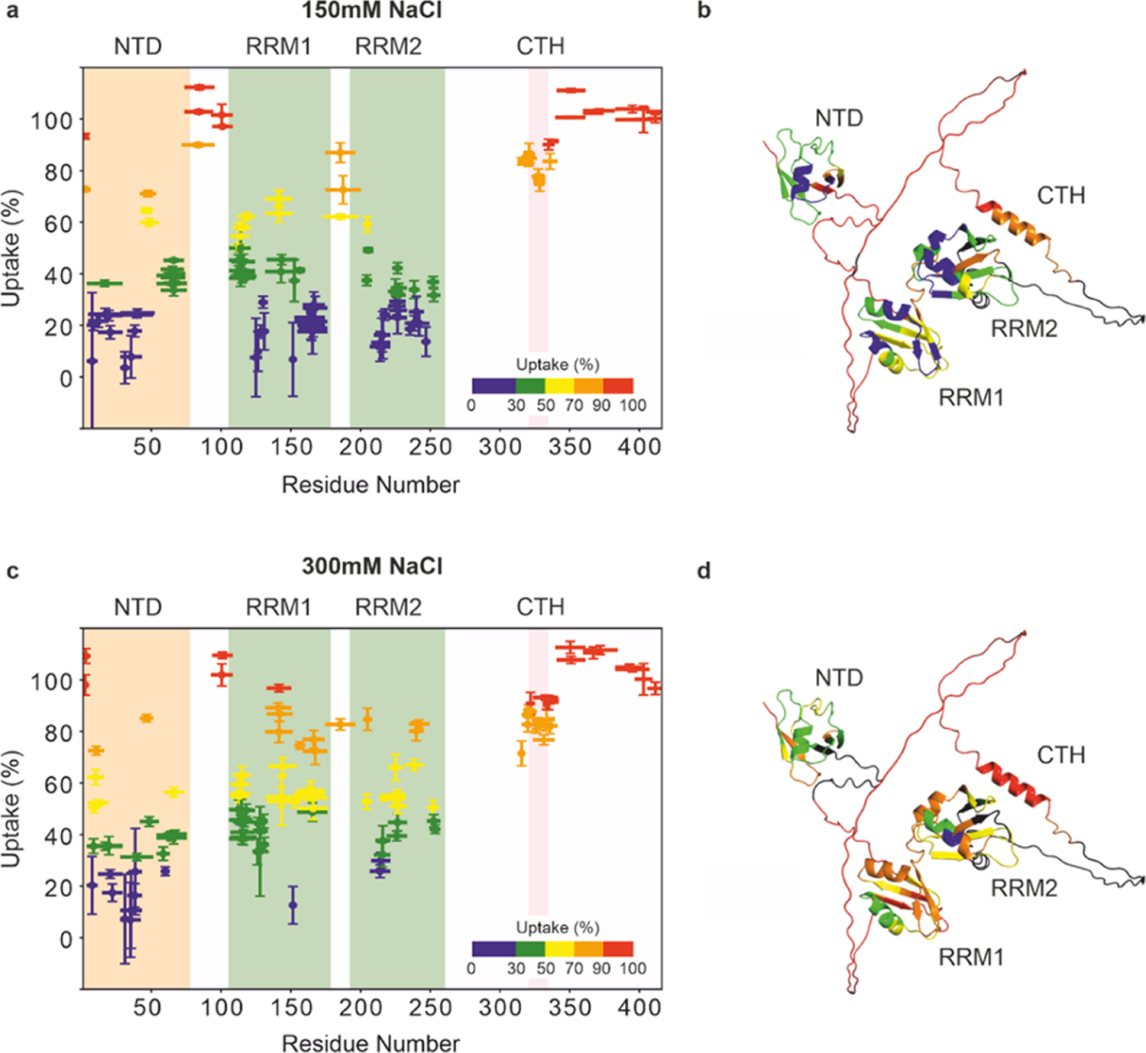
HDX-MS reveals dynamic disorder in monomeric TDP-43 and salt-dependent changes in conformational dynamics. (a, c) Percentage deuterium uptake of peptides from TDP-43 in (a) 150 mM NaCl or (c) 300 mM NaCl containing buffers (see the Methods section). Data were obtained by measuring the uptake of deuterium after a 30 s incubation and comparing this to the extent of deuterium incorporation after reaching maximal exchange. Note that because both pH and salt concentration can influence the rate of exchange, a maximally deuterated control was performed for each condition to correct for this and enable direct comparison between different buffers (indeed, we observe that in 300 mM NaCl containing buffer, the maximal relative fractional uptake values measured are lower than in 150 mM NaCl containing buffer, with values over all detected peptides of 42.65 ± 12.4 and 56.96% ± 10.5%, respectively; error represents the standard deviation over all measured peptides in each state, supporting our use of a normalization strategy to compare between buffer conditions). A line indicates the protein region spanned by the detected peptide, and data are shown as mean ± standard deviation of three replicate measurements. Peptides are colored by their percentage uptake values (see legend, inset). The positions of the folded domains in TDP-43 are indicated by the shaded areas (see Figure 1). (b, d) Percentage deuterium uptake values plotted on the AF2 model structure for TDP-43 in (b) 150 mM NaCl or (d) 300 mM NaCl containing buffers (see the Methods section). Note that residues are colored according to the percentage uptake value of the peptide comprising that residue that has the highest measured value.

First, we sought to interrogate the structure and dynamics of monomeric TDP-43 using HDX-MS. Given our desire to study the monomeric form of the protein, without our data being confounded by effects from aggregation during our analyses, we chose to study TDP-43 fused with a C-terminal maltose binding protein (MBP) tag for all of our HDX-MS experiments (TDP-43-MBP), as this allowed isolation of monomeric TDP-43-MBP by size exclusion chromatography (see the Methods section).^48^ To determine regions of protection from exchange, i.e., regions of secondary structure and/or intraprotein hydrogen bonding, we compared the extent of deuterium incorporation after a rapid labeling pulse (30 s) to a fully deuterium-labeled sample (see the Methods section) (Figure 2a). As expected, the folded NTD, RRM1, and RRM2 domains experienced the lowest extent of deuterium incorporation after the short labeling pulse, and we observed that regions of the protein that reached maximal uptake levels by 30 s were localized predominantly to the disordered regions that connect the folded domains of TDP-43. We have visualized these data on the AlphaFold 2 (AF2) predicted structure of the protein^52,53^ (Figure 2b). The C-terminal helical region (CTH) of the LCD has a lower extent of deuterium incorporation compared to the observed surrounding regions of the LCD (Figure 2a,b), suggesting that under physiological conditions some helicity is present, consistent with data from nuclear magnetic resonance (NMR) spectroscopy.^36,54^

We then correlated the extent of protection from exchange to the predicted local-distance difference test (pLDDT) generated by AF2, as a metric for disorder^55^ (Supporting Figure 3). From this analysis, it was evident that there was clustering of pLDDT/uptake values for peptides from the structured domains, the LCD, the CTH, and flexible linkers in-between domains. Peptides containing loops within the structured domains show higher deuterium uptake at 30 s than would be expected by the pLDDT score, but this likely represents the more dynamic nature of these features that are not captured by the pLDDT parameter from AF2. This is consistent with previous reports correlating metrics from AF2 with exchange kinetics from HDX,^56^ and demonstrates the power of HDX-MS to probe for predicted regions of order/disorder and transient structural elements in proteins.

Given the importance of electrostatic interprotein interactions in tuning the conformational landscape of disordered proteins,^42^ and the role of salt and buffer conditions in tuning LLPS and aggregation,^43,44^ we extended our HDX-MS analysis of monomeric TDP-43 further to examine the impact of higher salt concentrations (300 mM NaCl) [which increase the propensity of full length TDP-43^57^ and the CTD of TDP-43^35,58,59^ to both aggregate and undergo LLPS], on full length TDP-43 structure and dynamics. Given that intrinsic rates of deuterium exchange are buffer, salt and pH dependent,^60−62^ we performed further fully deuterated control experiments in buffer containing 300 mM NaCl and calculated the extent of deuterium incorporation in TDP-43 at a 30 s time point in this buffer. It is important to note that both pH and salt concentration can influence the rate of exchange^61,63^ and, therefore, fully deuterated controls were performed for each condition to correct for buffer effects and to enable direct comparison between the extent of deuterium uptake in different buffers. When comparing the extent of exchange in TDP-43 in solutions containing either 150 mM NaCl (Figure 2a,b) and 300 mM NaCl (Figure 2c,d), a clear increase in deuterium uptake in the structured domains was observed at the elevated NaCl concentration. This suggests that intraprotein hydrogen bonds within and/or between the folded domains are destabilized when the NaCl concentration is increased from 150 to 300 mM. Furthermore, the peptides containing the core of the C-terminal α-helix show a subtle increase in deuterium incorporation, however, this region of the protein remains more protected from exchange compared with peptides that span the rest of the LCD domain. Overall, these HDX-MS data confirm that under the buffer conditions used here, the helical region of the CTD is protected from exchange relative to the rest of the LCD, consistent with the presence of ordered structure, supporting the AF2 model of TDP-43 along with other structural data.^35−37^ Further, these data demonstrate that increasing the salt concentration modulates the dynamics of monomeric TDP-43, resulting in a structure that is globally more solvent exposed/less intraprotein hydrogen bonded.

### TDP-43 Aggregation is Modulated by Salt Concentration and RNA Binding in a Sequence-Specific Manner

Given that (i) the binding of TDP-43 to RNA has been shown previously to inhibit TDP-43 aggregation,^41^ (ii) added NaCl increases the propensity of full length TDP-43^57^ and the CTD of TDP-43^35,58,59^ to both aggregate and undergo LLPS, and, (iii) we identified by HDX-MS that salt modulates TDP-43 structural dynamics (Figure 2), we were interested to understand the interplay between these agonistic/antagonistic effects on LLPS/aggregation and the architecture of TDP-43-RNA assemblies. TDP-43 is known to bind to UG rich RNA sequences, and cross-linking and immunoprecipitation (CLIP) experiments have identified a 34 nucleotide sequence from the 3′-UTR of the TDP-43 mRNA that it binds to, called CLIP34NT.^19,20,26^ Therefore, we sought to examine the ability of CLIP34NT and a UG repeating sequence, UG(6) to bind to TDP-43 and tune its aggregation.

First, we sought to understand the affinities of the interactions of TDP-43 with the RNA oligonucleotides that we selected, CLIP34NT and UG(6), using microscale thermophoresis (MST). We focused our investigation on two concentrations of NaCl (150 and 300 mM) in light of our observation of a structural change in monomeric TDP-43 between these two solution conditions (Figure 2) by HDX-MS. In buffer containing 150 mM NaCl, both CLIP34NT and UG(6) bind to TDP-43 with similar EC_50_ values (383 and 355 nM respectively; Supporting Figure 4 and Supporting Table 1). This is in agreement with other published measurements of affinities for CLIP34NT and UG(6) using similar techniques.^40,64^ Notably, increasing the concentration of NaCl from 150 to 300 mM has no impact on the affinity of UG(6) for TDP-43 (355 nM at 150 mM NaCl and 373 nM at 300 mM NaCl), whereas the affinity of CLIP34NT for TDP-43 decreases markedly (383 nM at 150 mM NaCl and 1169 nM at 300 mM) (Supporting Figure 4 and Supporting Table 1).

It is important to note that none of the binding curves could be fitted using a simple a 1:1 interaction model. This is because TDP-43 has two RRM domains connected by a highly flexible linker, each of which contain two distinct RNA binding motifs, called RNP-1 and RNP-2, and therefore each TDP-43 monomer has multiple binding sites for RNA.^65^ As a result, a variable slope fitting model was used to fit the data, and the Hill coefficient values for all binding curves were >1 (Supporting Figure 4 and Supporting Table 1), consistent with multiple RNA binding motifs being involved in cooperative binding the oligonucleotides and/or the formation of higher order assemblies. For both conditions tested UG(6) had higher Hill slope values (∼5 for UG(6) and ∼3.6 for CLIP34NT) (Supporting Table 1) suggesting higher cooperativity in the binding of the UG repeats despite the increased length of CLIP34NT. Further, the Hill coefficient for UG(6) binding is not effected by increasing the NaCl concentration, whereas the fitted value for CLIP34NT is reduced (Supporting Table 1), suggesting that elevated NaCl levels have an effect on the cooperativity/valency of the interaction between TDP-43 and CLIP34NT.

**Figure 3:**
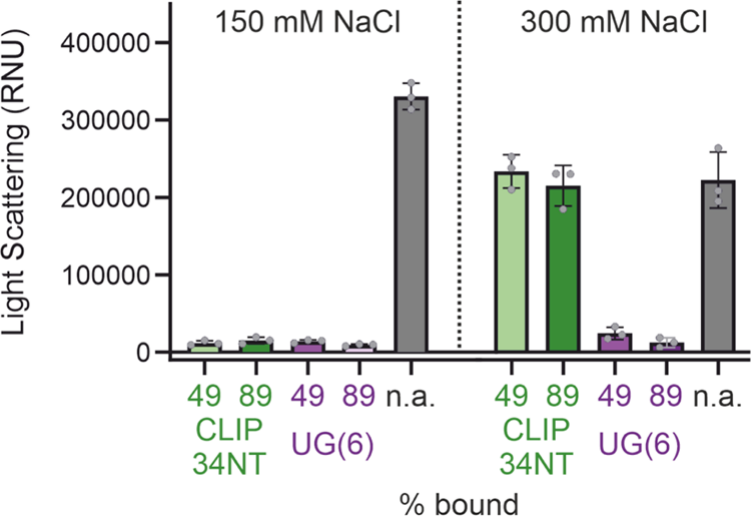
Interplay of RNA oligonucleotide sequence and NaCl concentration on the antagonization of TDP-43 aggregation. Addition of CLIP34NT or UG(6) to TDP-43 in either 150 or 300 mM NaCl solutions have different effects on aggregation. Light scattering measurements were taken after 6 h incubation of TDP-43 aggregation at different concentrations of CLIP34NT and UG(6) to achieve different degrees of protein saturation by RNA. Note that the extent of protein saturation was calculated based on affinity measurements to TDP-43-MBP, and nephelometry measurements used MBP-cleaved TDP-43. Kinetic data can be seen in Supporting Figure 5. See Supporting Figure 4 and Supporting Tables 1 and 2 for binding data used to calculate the degrees of saturation. Bars indicate the mean of three independent experiments, and the error bar shows the standard deviation. Individual data points are shown. The light scattering measurements for a TDP-43 solution without addition of RNA is shown as a positive control. RNU = relative nephelometry units.

With the measured EC_50_ values in-hand, we next set out to test the ability of our different RNA oligonucleotide sequences to prevent TDP-43 aggregation under high and low salt conditions. To enable direct comparison between the ability of the different RNA sequences to prevent aggregation upon binding, we performed experiments under conditions where the amount of TDP-43 bound to each RNA sequence being studied was comparable and started by testing conditions where ∼89% protein saturation was achieved. Interestingly, we observed that the ability of CLIP34NT to impair TDP-43 aggregation in the presence of 150 mM NaCl is ablated when the NaCl concentration is raised to 300 mM (Figure 3, Supporting Figure 5). In the presence of UG(6) we observe that this RNA does prevent aggregation in the presence of 300 mM NaCl (Figure 3, Supporting Figure 5). For the HDX-MS experiments that we used to probe the RNA-bound state of TDP-43 (see below), we used conditions where the protein was ∼49% RNA bound, and therefore, we performed control experiments to determine if lower RNA concentrations resulted in different effects on RNA-mediated aggregation inhibition. The data from these experiments show that when TDP-43 was ∼49% RNA bound the inhibitory effects of RNA-binding were comparable to conditions where ∼89% protein saturation was achieved (Figure 3, Supporting Figure 5).

As a control, we performed affinity and aggregation measurements with UG(17), an RNA oligonucleotide of the same length as CLIP34NT. This sequence behaved similarly to UG(6), in that its affinity for TDP-43 and ability to prevent TDP-43 aggregation was comparable at both 150 mM and 300 mM NaCl (Supporting Figure 6, Supporting Table 1). However, our data do show that similarly to CLIP34NT, which is the same length as UG(17), a reduction in the fitted Hill coefficient occurred when the NaCl concentration was increased. This is dissimilar to UG(6), where the Hill coefficient was unaffected by increasing the concentration of NaCl, and suggests that the cooperativity of the binding interaction between TDP-43 and RNA is dependent on RNA sequence length. Taken together, these data suggest that the prevention of TDP-43 aggregation by RNA is dependent both on environmental conditions (here NaCl concentration) and the sequence of the RNA oligonucleotide that is bound.

### HDX-MS Suggests a Mechanism for RNA-Mediated Aggregation Inhibition

**Figure 4:**
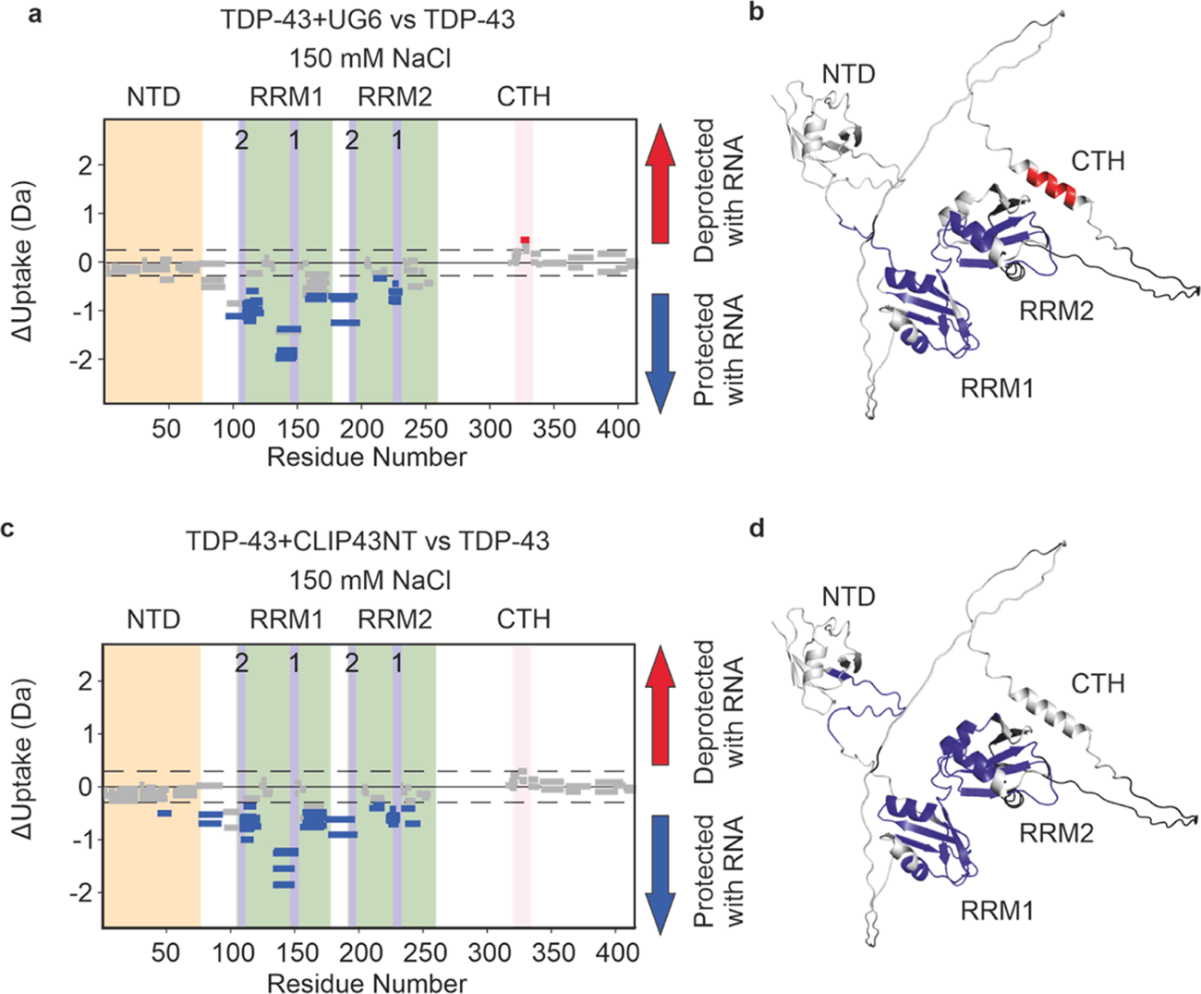
RNA binding to TDP-43 probed by HDX-MS. Wood’s plots showing the difference in deuterium uptake in TDP-43 in 150 mM NaCl-containing buffer at a 2 min HDX time point, comparing TDP-43 alone with TDP-43 in the presence of (a) UG(6) or (c) CLIP34NT. Wood’s plots were generated using Deuteros 2.0. Peptides colored in blue or red, respectively, are protected or deprotected from exchange in the presence of CLIP43NT/UG(6). The NTD (orange), RRMs (green) and CTH (pink) are indicated as shaded regions. RNP-1 and RNP-2 motifs in RRM1 and RRM2 are labeled and indicated by purple shading. Peptides with no significant difference between conditions, determined using a hybrid significance test^67^ with a 98% confidence interval are shown in gray. Note that the hybrid significance test comprises two components: a *t* test at the peptide level and an estimated global significance cutoff based on an estimation of the experimental error (see ref (67)), indicated here by the dotted line. To meet the criteria for significance, each peptide must pass both tests, and therefore some peptides which lie outside the global significance cutoffs are not statistically significantly different. Such a strategy has been reported to reduce the risk of false positives.^67^ (b, d) AF2 structure of TDP-43 with regions of TDP-43 protected or deprotected in the presence of (b) UG(6) or (d) CLIP34NT colored in blue or red, respectively. See the Methods section for experimental details. Wood’s plots for other HDX time points are shown in Supporting Figure 7.

We next sought to dissect the impact that the binding of RNA has upon the global conformation of full length monomeric TDP-43 using differential HDX-MS. We first compared the uptake of deuterium in the presence or absence of UG(6) in buffer containing 150 mM NaCl (Figure 4a,b, Supporting Figure 7). In the presence of UG(6), protection from exchange was observed within the two RRM domains. Peptides that were protected from exchange in the presence of RNA span the canonical RNP-1 and RNP-2 binding motifs located within these domains (residues 106–112 and 145–152 in RRM1, and residues 193–198 and 227–234 in RRM2^64−66^). Significant protection from exchange was also identified in peptides that encompass the residues Arg171, Lys176, Asp174, Lys176, where mutations to Ala have been previously shown to reduce RNA binding affinities up to 20-fold.^64^ Together, this suggests that the protection from exchange in these regions of the protein that are key for nucleic acid recognition was due to direct interactions with RNA.

We also observed protection from exchange in peptides spanning residues 236–248 and 249–256, which contain the ^247^DLIIKGISVHI^257^ segment, found in RRM2, that has been shown to form amyloid fibrils *in vitro*,^68^ but lies outside of the amyloid core as defined by solved structures of TDP-43 filaments (which tend to comprise residues ca. 280–360).^11,25,69−71^ This region is predicted to be amyloid prone using various *in silico* aggregation and amyloid predictor servers (see ref (68), and Supporting Figure 8). Further to this, it has been shown, using NMR spectroscopy that upon RNA binding, Asp247, which is found in this region of RRM2, forms a salt bridge with the RRM1 RNP1 residue Arg151.^72^ Notably, in the presence of RNA, we observe protection in the regions of the protein that comprise the key residues of this inter-RRM domain salt bridge (Figure 4). This observation, along with protection from the exchange we observed in the linker residues between RRM domains is consistent with data suggesting that RNA binding promotes association of the RRM domains.^73^

The regions of protection from HDX identified in the RRM domains of TDP-43 in the presence of both UG(6) (Figure 4a,b) and CLIP34NT (Figure 4c,d) are comparable under conditions where the percentage of TDP-43 that is bound is the same (∼49% bound), suggesting a similar conformation is adopted by the RRM domains on binding to these two substrates. However, to interrogate the similarities/differences between the bound states, we performed an additional quantitative analysis (Supporting Figure 9). Interestingly, we found that peptides covering the RNP-2 motif on RRM1 show significant protection from deuterium uptake in the UG(6)-bound state compared to CLIP34NT bound state at the 0.5 and 2 min HDX time points, but not at the 5 min time point. This suggests that the extent of protein-RNA hydrogen bonding at the RNP-1 motif in RRM1 is enhanced when UG(6) binds compared to CLIP34NT, and demonstrates that HDX-MS can provide evidence of RNA sequence-dependent selectivity in the binding mode of RNA oligonucleotides to proteins, even when the RNA oligonucleotides have comparable affinities.

**Figure 5:**
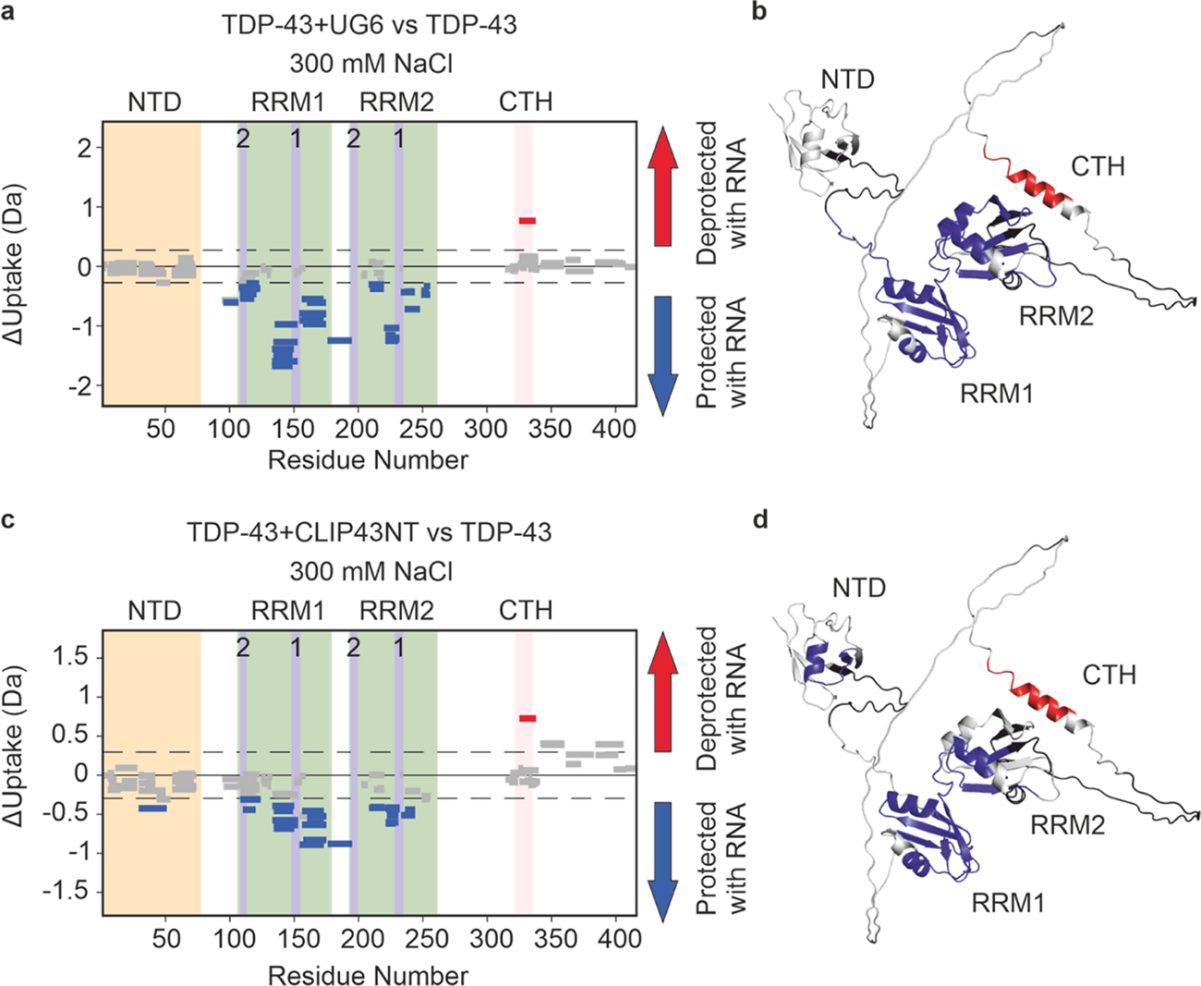
RNA binding to TDP-43 at an elevated NaCl concentration probed by HDX-MS. Wood’s plots showing the difference in deuterium uptake in TDP-43 in 300 mM NaCl-containing buffer at a 2 min HDX time point, comparing TDP-43 alone with TDP-43 in the presence of (a) UG(6) or (c) CLIP34NT. Wood’s plots were generated using Deuteros 2.0. Peptides colored in blue or red, respectively, are protected or deprotected from exchange in the presence of CLIP43NT/UG(6). The NTD (orange), RRMs (green) and CTH (pink) are indicated as shaded regions. RNP-1 and RNP-2 motifs in RRM1 and RRM2 are labeled and indicated by purple shading. Peptides with no significant difference between conditions, determined using a hybrid significance test^67^ with a 98% confidence interval are shown in gray. Note that the hybrid significance test comprises two components: a *t* test at the peptide level and an estimated global significance cutoff based on an estimation of the experimental error (see ref (67)), indicated here by the dotted line. To meet the criteria for significance, each peptide must pass both tests, and therefore some peptides which lie outside the global significance cutoffs are not statistically significantly different. Such a strategy has been reported to reduce the risk of false positives.^67^ (b, d) AF2 structure of TDP-43 Regions of TDP-43 protected or deprotected in the presence of (b) UG(6) or (d) CLIP34NT colored in blue or red, respectively. See the Methods section for experimental details. A complete set of Wood’s plots for all other HDX time points recorded are shown in Supporting Figure 10.

We then examined the same interactions under the high salt buffer conditions (300 mM NaCl). Binding to both RNA oligonucleotides resulted in significant protection in peptides containing all 4 RNP motifs along with additional peptides in the RRM1 (160–173/5, 176–197), and RRM2 domains (249–256) (Figure 5, Supporting Figure 10).

**Figure 6:**
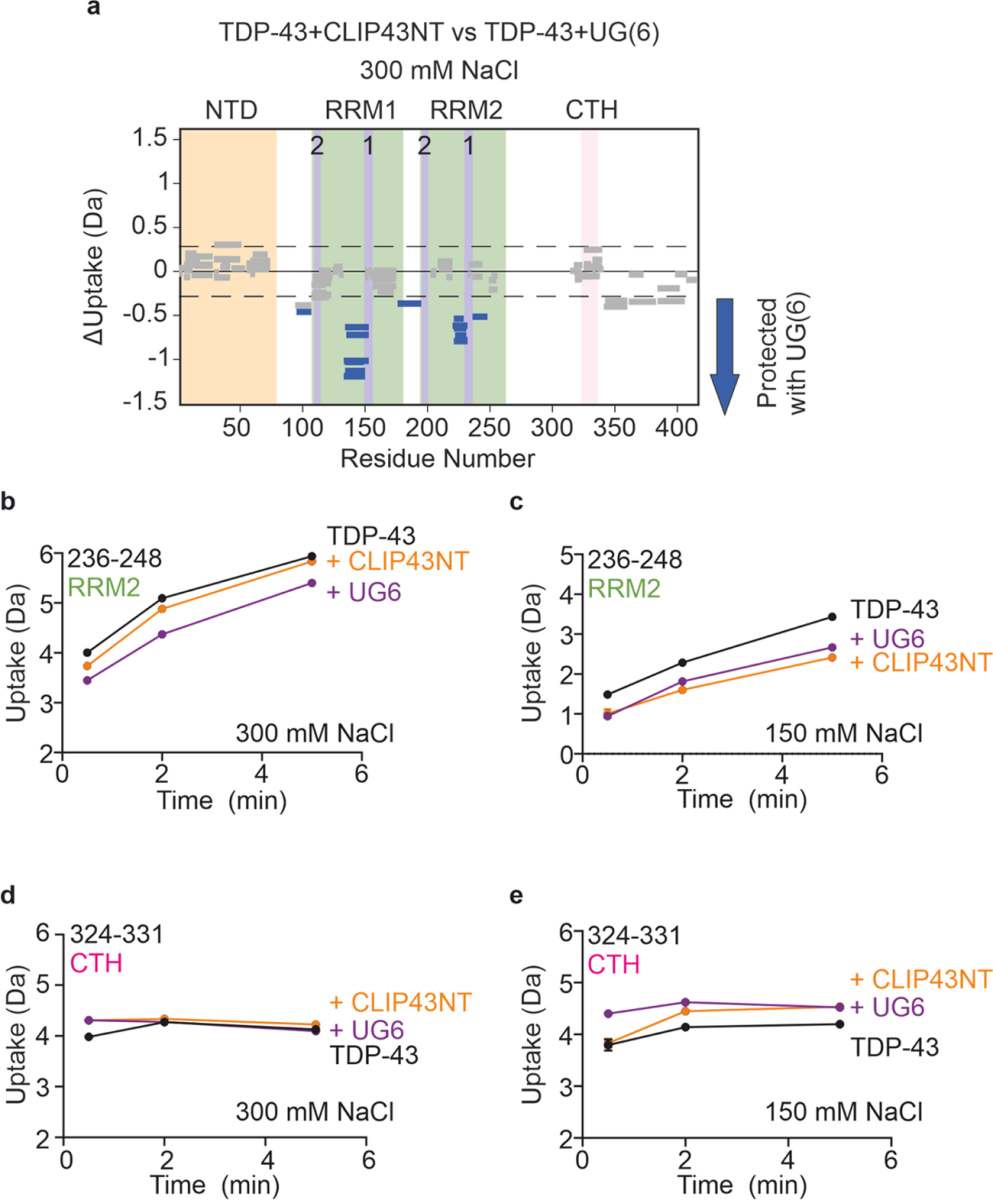
Comparison of the RNA-bound states of TDP-43 reveals distinct mechanisms of RNA binding/recognition. (a) Wood’s plots showing the difference in deuterium uptake in TDP-43 in 300 mM NaCl-containing buffer at a 2 min HDX time point, comparing TDP-43 bound to CLIP43NT and TDP-43 bound to UG(6). Wood’s plots were generated using Deuteros 2.0. Peptides colored in blue are protected from exchange in the presence of UG(6) relative to the presence of CLIP34NT. The NTD (orange), RRMs (green) and CTH (pink) are indicated as shaded regions. RNP-1 and RNP-2 motifs in RRM1 and RRM2 are labeled and indicated by purple shading. Peptides with no significant difference between conditions, determined using a hybrid significance test^67^ with a 98% confidence interval are shown in gray. Note that the hybrid significance test comprises two components: a *t* test at the peptide level and an estimated global significance cutoff based on an estimation of the experimental error (see ref (67)), indicated here by the dotted line. To meet the criteria for significance, each peptide must pass both tests, and therefore some peptides which lie outside the global significance cutoffs are not statistically significantly different. Such a strategy has been reported to reduce the risk of false positives.^67^ (b, c) Deuterium uptake plots for a peptide from the RNP-1 motif in RRM2 in the absence/presence of CLIP34NT or UG(6). Deuterium exchange was conducted in buffer containing either (b) 150 mM NaCl or (c) 300 mM NaCl. (d, e) Deuterium uptake plots for a peptide from the CTH in the absence/presence of CLIP34NT or UG(6). Deuterium exchange was conducted in buffer containing either (d) 150 mM NaCl or (e) 300 mM NaCl. Data are plotted as the mean value from three replicate measurements, and error bars on deuterium uptake plots represent the standard error of the mean (*n* = 3). In most cases, the standard error is smaller than the size of the data point shown, so cannot be observed in the figure.

To understand the differences in how the two RNAs engage TDP-43 under high salt conditions, i.e., conditions where they have differential effects on preventing TDP-43 aggregation, the two bound states were compared (Figure 6a, Supporting Figure 11). The extent of deuterium uptake in peptides spanning the RNP-2 motifs on both RRM1 and RRM-2 were not significantly different when bound to the two different RNAs. However, both RNP-1 motifs were significantly protected in the presence of UG(6) compared to the presence of CLIP34NT. Notably, the peptide containing Asp247 experienced greater protection from exchange in the presence of UG(6) compared with CLIP34NT (n.b. the difference between the uptake of this peptide unbound vs the CLIP34NT bound state was not statistically significant, but the difference was significant upon binding UG(6)) (Figure 6b) (hybrid significance test, *p* < 0.02). Additionally, in the presence of 150 mM NaCl, comparable levels of protection from exchange were observed in this peptide when bound to both RNA oligonucleotides (Figure 6c). We also observed protection from exchange in the linker region between RRM and RRM2 when bound to UG(6) compared to when bound to CLIP34NT (Figure 6a). Moreover, the most significant difference in deuterium uptake between the two RNA bound states was within a region of the protein that includes Arg151 (Figure 6a), which is involved in the formation of the interdomain salt bridge between RRM domains,^72^ suggesting that the interdomain interactions between RRM domains, mediated by this salt bridge, are disturbed in the high salt buffer upon binding CLIP34NT but not when binding UG(6). Combined, this HDX-MS analysis suggests that different RNA sequences differentially engage with the RNA binding motifs when bound to TDP-43.

Another interesting feature of our HDX-MS data is that upon RNA binding, in all conditions (but not all time points), a distinct patch of deprotection was detected at the site of the CTH located in the LCD of TDP-43 (Figure 6d,e). This suggests that RNA binding may be destabilizing the structured CTH in this region or be destabilizing intramolecular contacts involving this sequence. This region is a major site of disease-associated mutations, and it is known that mutations in this region that disrupt or enhance α-helicity can impact the preference of TDP-43 to bind different RNA sequences.^10,36^ Moreover, it has been reported that nucleic acids can interact with specific Arg and Lys residues in the LCD,^74^ but no protection from HDX was observed in our experiments, which would be expected for binding events. However, it should be noted that no sequence coverage was obtained for a significant portion of the LCD due to low sequence complexity (Supporting Figures 1 and 2), and hence no information can be obtained for these regions. Additionally, the time scale of our measurements (sec-min) may be too long to capture differences in HDX, especially for disordered regions where more rapid exchange experiments (i.e., on the msec time scale) may be needed.^62,75,76^

Additionally, there was significant protection observed in segments of the N-terminal domain upon RNA binding at some HDX time points. When bound to CLIP34NT, significant protection from exchange was observed in peptides from the N-terminal domain for the 2 min time point in 150 mM NaCl containing buffer, and for the 2 and 5 min time points in 300 mM NaCl. When bound to UG(6), significant protection from exchange was only observed at the 5 min time point in the presence of 150 mM NaCl. No significant protection from exchange was observed in 300 mM NaCl-containing buffer (Figures 4 and 5, Supporting Figures 7 and 10). Given that the NTD has not been implicated directly in RNA binding,^39,77,78^ this suggests further allosteric changes upon RNA binding. This is consistent with evidence that a small molecule that binds the NTD allosterically impacts binding at the RRM domains of TDP-43.^79^

In summary, using HDX-MS combined with biochemical characterization, we identify similarities and differences between RNA oligonucleotide engagement by TDP-43 in the presence of different RNA sequences, and provide evidence that the mechanism of RNA binding is dependent on both RNA sequence and NaCl concentration.

## Discussion

RNA binding by TDP-43 is not only fundamental to its biological function but also in regulating its propensity to aggregate and undergo LLPS.^4,26,41^ It has been widely reported that aggregation of TDP-43 is inhibited upon binding RNA,^27,41,79,80^ but the mechanism underpinning this protective effect is not fully understood. Here, we have interrogated the impact of binding of model RNA molecules on full length TDP-43. We found that aggregation can be inhibited by the addition of two well-characterized TDP-43 binding oligonucleotides, UG(6) and CLIP34NT, at near physiological NaCl concentrations (150 mM).^81^ However, elevated NaCl levels (300 mM) reduced the affinity of CLIP34NT for TDP-43 and abolished the inhibition of aggregation by this RNA that is observed under more physiological salt concentrations.

Evidence from previous studies suggests that RRM1 is the major driver of binding affinity to RNA molecules and that RRM2 alone has a much lower affinity to RNA,^32^ although it has been shown that there is cooperativity in the binding of RRM domains to RNA.^47,78^ This is consistent with the data from HDX-MS that we present here, as the biggest differences in uptake between the two RNA oligonucleotide bound states in high salt conditions are observed in RRM1 (Figure 6a). This suggests that altered interactions of RNA occur with this domain depending on their length/sequence, despite the experiments being conducted under conditions where the same proportion of TDP-43 was bound to RNA. Further, the effects in our HDX-MS experiments on the NTD and CTH that we detected in the presence of RNA raise additional questions for understanding allosteric modulation of TDP-43 via RNA binding at the RRM domains. For example, it remains unclear precisely which TDP-43 interdomain contacts are populated significantly in solution, how these contacts change when bound to different RNA sequences (including mRNA) and how solution conditions tune these interactions.

Nevertheless, data from molecular dynamics simulations have suggested that in the presence of RNA, fewer interdomain contacts in TDP-43 are populated compared to when RNA is absent.^73^ This finding, combined with our data showing that the linker region between RRM1 and RRM2 is more protected from exchange when bound to UG(6) compared to CLIP34NT (Figure 6), suggests an RNA sequence dependent effect on these interdomain interactions. Evidence from NMR spectroscopy has indicated that a salt bridge involving Asp247 in RRM2 and Arg151 of RRM1 is stabilized upon RNA binding.^72^ Indeed, alanine substitution of these two residues has been shown to reduce the affinity of the TDP-43 RRMs to UG rich RNA by >37 fold, suggesting that interdomain interactions between RRM1 and RRM2 are involved in regulating TDP-43 RNA binding function.^64^ Intriguingly, the peptides involving the residues of this salt bridge are more protected from exchange when bound to UG(6) compared to when bound to CLIP43NT under nonphysiological NaCl levels (Figure 6), suggesting that this region is more occluded from solvent when bound to UG(6), possibly because of disruption of this salt bridge when bound to CLIP34NT. Given that Asp247 lies within an amyloidogenic region of RRM2 (see ref (68), and Supporting Figure 8), and that under the conditions measured CLIP34NT no longer prevents TDP-43 aggregation, this suggests that there could be a role for interdomain interactions in preventing the exposure of aggregation-prone regions buried in RRM2. However, the role (if any) of the RRM domains in mediating TDP-43 aggregation is poorly understood,^47,82−84^ and exposure of such an aggregation-prone region to act as a template for TDP-43 self-assembly may also require (local) protein unfolding.

Evidence suggests that an intermediate state is present on the RRM2 unfolding pathway, but the role for these non-native states in mediating TDP-43 aggregation remains undetermined.^82,83,85,86^ Further to this, data from thermal and chemical denaturation experiments have shown that the isolated RRM2 domain is unusually stable, but the tethered RRM1-RRM2 construct is destabilized,^65,82^ suggesting that coupling of the domains results in a higher propensity to unfold. Combined, this highlights the need to elucidate the stabilities and unfolding propensities of the domains of TDP-43 in the context of the full-length protein. Such an understanding may help to uncover the structural mechanism by which specific RNA sequences modulate TDP-43 aggregation, potentially by influencing RRM domain stability, and could reveal a role for the aggregation-prone region found in RRM2 in the aggregation mechanism of TDP-43.

It is important to note that although the aggregation-prone region in RRM2 has been shown to be capable of forming amyloid fibrils in isolation , it has not been identified in the ordered structural core of TDP-43 amyloid fibrils, including fibrils isolated from patients.^11,68−70^ There is evidence from many other amyloidogenic systems for a role of flanking regions, that are ultimately not found in the fibril core, in tuning the propensity for protein self-assembly.^71,87,88^ Moreover, truncated TDP-43 found in patient samples often comprises the second portion of RRM2, in addition to the LCD, suggesting that this amyloidogenic sequence in RRM2 may be a player in the molecular basis of TDP-43 proteinopathies.^83^

We hypothesize that the reason why UG(6) binding to TDP-43 is unaffected by increased NaCl levels, whereas CLIP34NT binding affinity is reduced when the NaCl concentration is raised, is because UG(6) better satisfies the proposed minimum consensus RNA sequence for TDP-43 binding.^64^ Our observation also has implications for identifying and characterizing modulators of LLPS and aggregation more generally, as data from *in vitro* screening could be confounded by the nonphysiological solution conditions that may be used to tune the phase behavior of proteins and promote LLPS/aggregation, and therefore identified binders/inhibitors may not be functional *in vivo*.

While HDX-MS can inform on protein solvent accessibility/dynamics/hydrogen bonding, it is not always possible to conclusively ascertain if a change in deuterium exchange is due to alterations in interprotein, intraprotein or, in our case, protein-RNA hydrogen bonding. Multiple copies of TDP-43 may be binding to CLIP34NT, as has been previously reported in studies utilizing the isolated RRM domains,^19,22,28^ whereas UG(6) has only been shown to accommodate one TDP-43 molecule.^40^ Our data are consistent with cooperative binding to both RNA sequences and while elevated NaCl levels reduce the fitted Hill coefficient for the interaction of TDP-43 with CLIP34NT and UG(17), there is no effect of elevated NaCl levels on the fitted Hill coefficient for UG(6) (Supporting Table 1). This suggests that the co-operativity/valency of the TDP-43-CLIP34NT binding event may be different under different salt conditions. This could be resulting in the decrease in TDP-43 affinity for CLIP34NT at elevated NaCl levels and may be influencing the measured levels of deuterium exchange of some peptides. A further point of note is that our HDX-MS and RNA affinity measurements were performed using monomeric MBP-tagged TDP-43. It is possible that the RNA affinity and structural dynamics of the protein may have been influenced by the presence of the MBP tag. However, this construct enabled us to probe the conformational landscape of monomeric TDP-43 and its assemblies with RNA without our data being confounded by aggregation, thereby providing a more nuanced understanding of how TDP-43 monomers adopt different structural states in response to nucleic acid binding, thus enabling us to understand how these structural features may correlate with protein functionality and aggregation propensity.

## Conclusions

Targeting protein aggregation remains a key goal for the treatment of neurodegenerative diseases and efforts in this field have focused on the targeted clearance of fibrillar protein aggregates.^89^ Recently, two monoclonal antibodies (mAbs; aducanumab and lecanemab), have become FDA approved drugs which target the amyloid protein amyloid-β (Aβ). Both mAbs showed marked reduction of Aβ plaques by positron emission tomography in patients who met the clinical criteria of Alzheimer’s disease.^90,91^ However, given the complexity of protein aggregation processes, an attractive therapeutic strategy is to stabilize the native state of aggregation-prone proteins.^92,93^ Here, our data are consistent with a model whereby interdomain contacts, mediated by specific RNA sequences, stabilize the native state of TDP-43 and prevent aggregation. However, we also identify that the interactions which stabilize these interdomain contacts are tunable and sensitive to the solution environment. This provides evidence that, in the future, it will be important to consider the precise cellular and subcellular solution environment if we are to successfully stabilize monomeric TDP-43, in efforts to develop this as a viable therapeutic strategy to treat TDP-43 associated proteinopathies.

## Data Availability

The raw HDX-MS data have been deposited to the ProteomeXchange Consortium via the PRIDE partner repository with the data set identifier PXD054930.

## Supporting Information Available

HDX sequence coverage maps; correlation of pLDTT with uptake; microscale thermophoresis binding curves; nephelometry data; additional Wood’s plots; data from aggregation prediction algorithms; measured binding affinities and HDX-MS data summary (PDF)
