# Supplementary material for: Hydrogen–Deuterium Exchange Mass Spectrometry Reveals Mechanistic Insights into RNA Oligonucleotide-Mediated Inhibition of TDP-43 Aggregation
Source: J Am Chem Soc. 2024 Nov 29;146(49):33626–39. doi: 10.1021/jacs.4c11229 (PMC11638948; doi:10.1021/jacs.4c11229)
Supplement: Supplementary file 1 — ja4c11229_si_001.pdf [file ja4c11229_si_001.pdf]

Supplementary Information

Hydrogen-deuterium exchange mass spectrometry reveals mechanistic insights into RNA oligonucleotide-mediated inhibition of TDP-43 aggregation

Thomas C. Minshull[†], Emily J. Byrd[†], Monika Olejnik[†], Antonio N. Calabrese[†*]

[†] Astbury Centre for Structural Molecular Biology, School of Molecular and Cellular Biology, Faculty of Biological Sciences, University of Leeds, Leeds, LS2 9JT, United Kingdom

* To whom correspondence should be addressed. a.calabrese@leeds.ac.uk

20mM HEPES,150mM NaCl pH 7.4

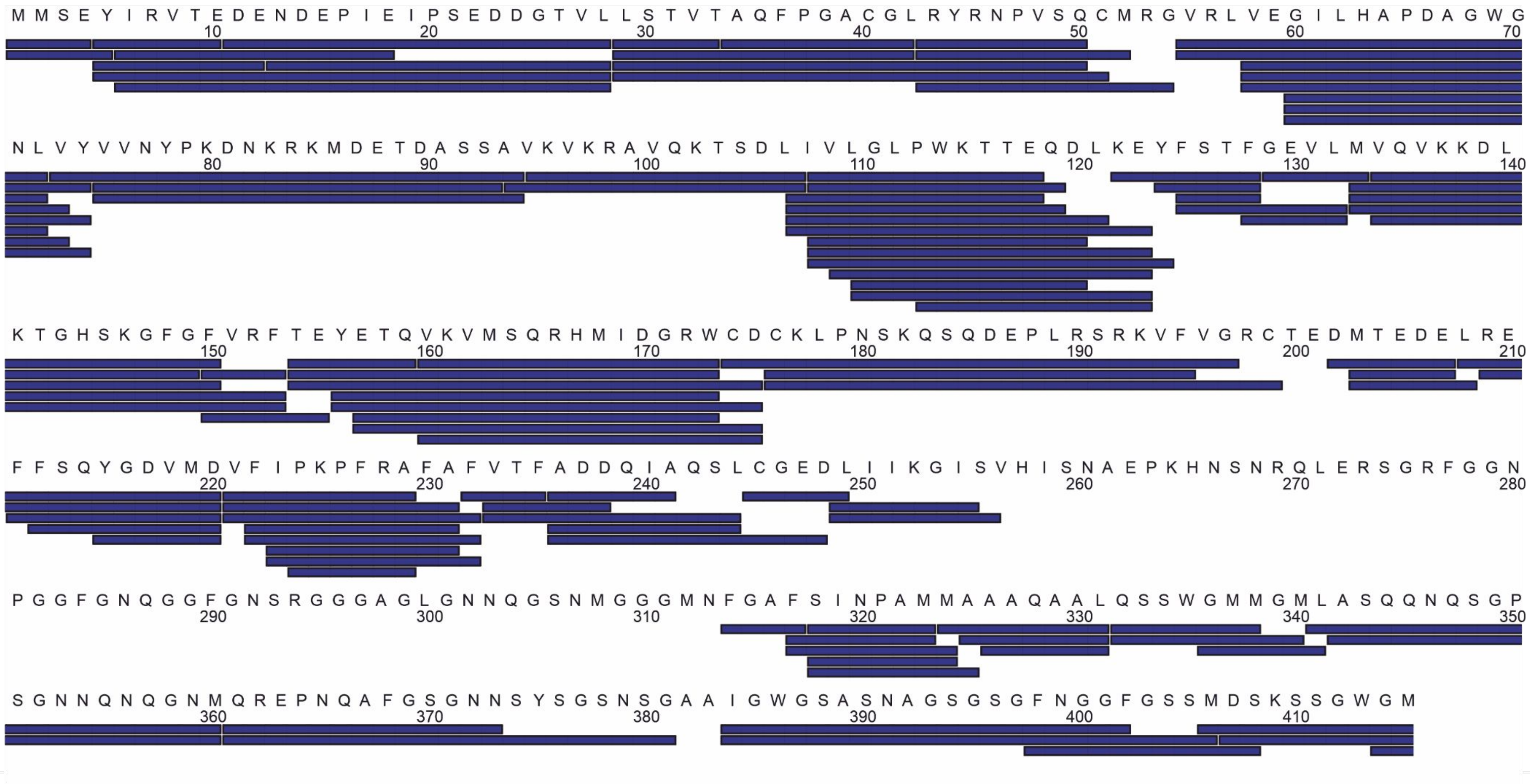

Supplementary Figure 1. Sequence coverage map of TDP-43 in HDX-MS experiments in 20 mM HEPES, 150 mM NaCl pH 7.4. Blue bars represent the individual peptides detected. See Supplementary Table 3 for additional details of HDX-MS parameters.

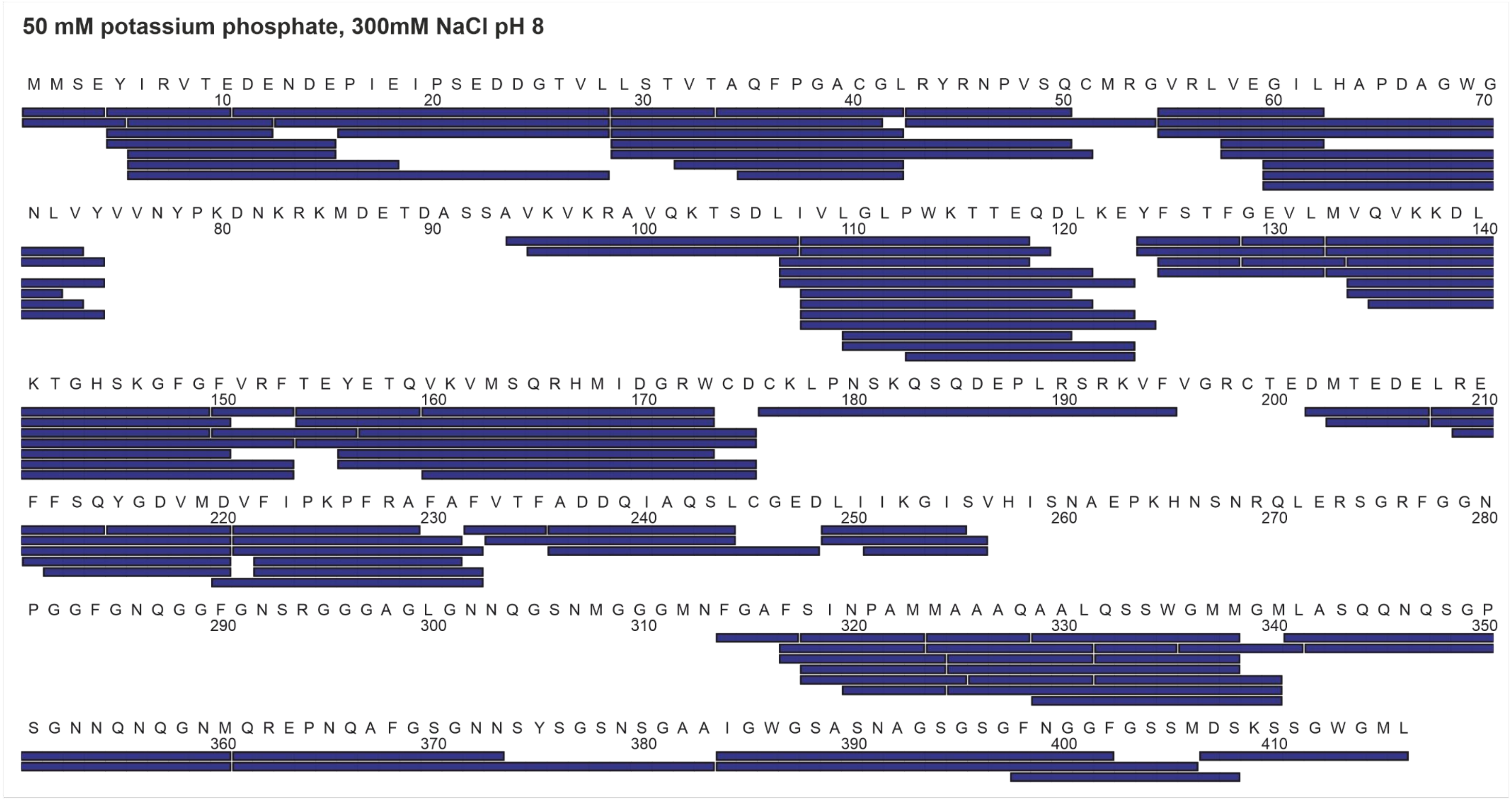

Supplementary Figure 2. Sequence coverage map of TDP-43 in HDX-MS experiments in 50 mM potassium phosphate, 300 mM NaCl, pH 8.0. Blue bars represent the individual peptides detected. See Supplementary Table 3 for additional details of HDX-MS parameters.

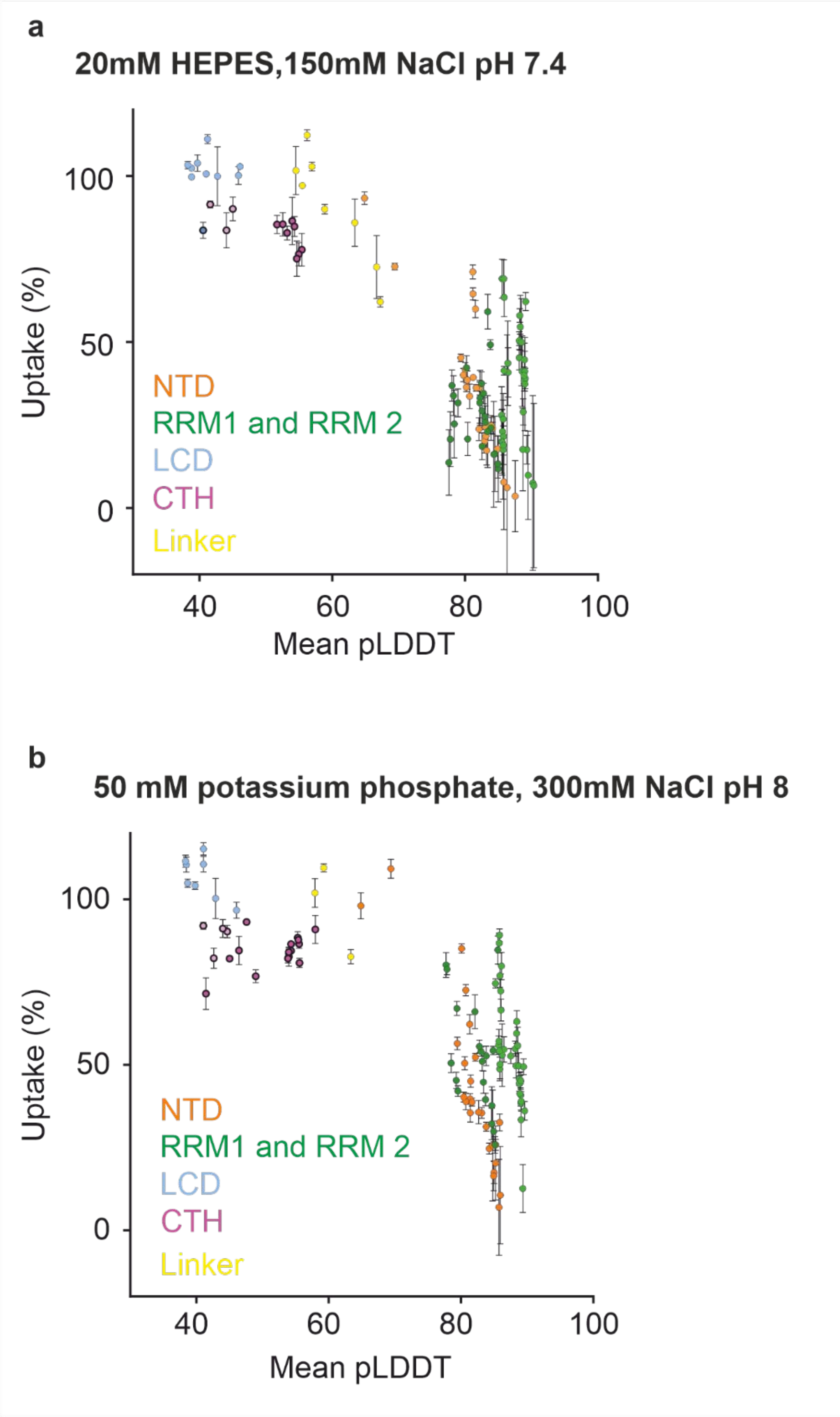

Supplementary Figure 3. Correlation of pLDTT with the extent of deuterium uptake. As the pLDTT parameter from AF2 provides a residue-level metric, for each peptide detected the mean pLDTT value from all residues was calculated. This value was plotted against the measured uptake value. Uptake data are shown as mean ± standard deviation of three replicate measurements. Datapoints are coloured according to their location in the TDP-43 structure (see Figure 1 in the main text). Peptides from the LCD (light blue) have the lowest mean pLDDT values and highest measured uptake, with peptides from the CTH clustering with lower uptake values and slightly higher pLDDT values. Peptides from the NTD, RRM1 and RRM2 domains have higher mean pLDDT values and are spread over a range of uptake values, consistent with domain architectures that comprise regions of differential protection/exposure from solvent.

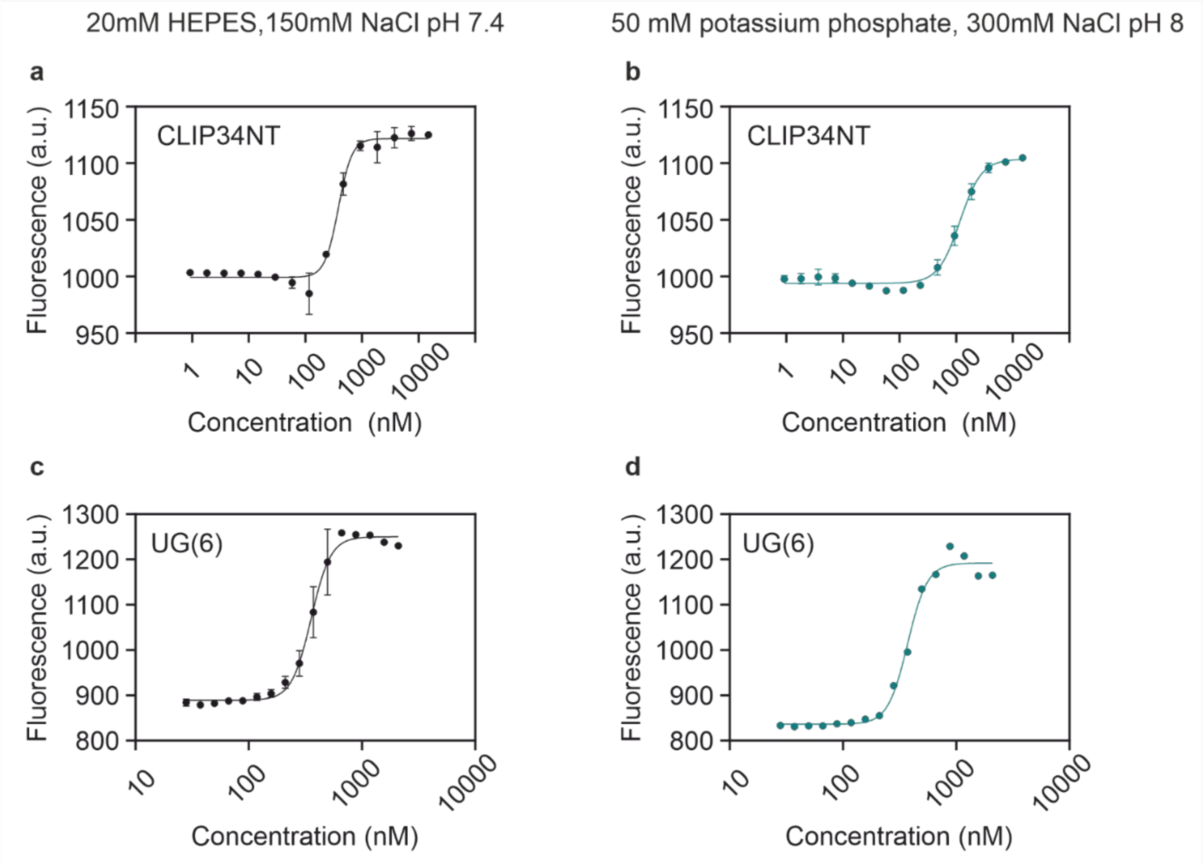

Supplementary Figure 4. MST binding curves for TDP-43 and RNA oligonucleotides under different buffer conditions. (a,b) MST binding curves for TDP-43 binding to CLIP34NT in **(a)** 20 mM HEPES 150 mM NaCl pH7.4 and **(b)** 50 mM potassium phosphate 300 mM NaCl pH 8.0. **(c,d)** MST binding curves for TDP-43 binding to UG(6) in **(c)** 20 mM HEPES 150 mM NaCl pH7.4 and **(d)** 50 mM potassium phosphate 300 mM NaCl pH 8.0.Two replicates were performed and averaged prior to fitting, and the error (standard deviation) between replicates is plotted. See methods for experimental details and details of fitting procedure. See **Supplementary Table 1** for fitted EC_{50} and Hill coefficient values.

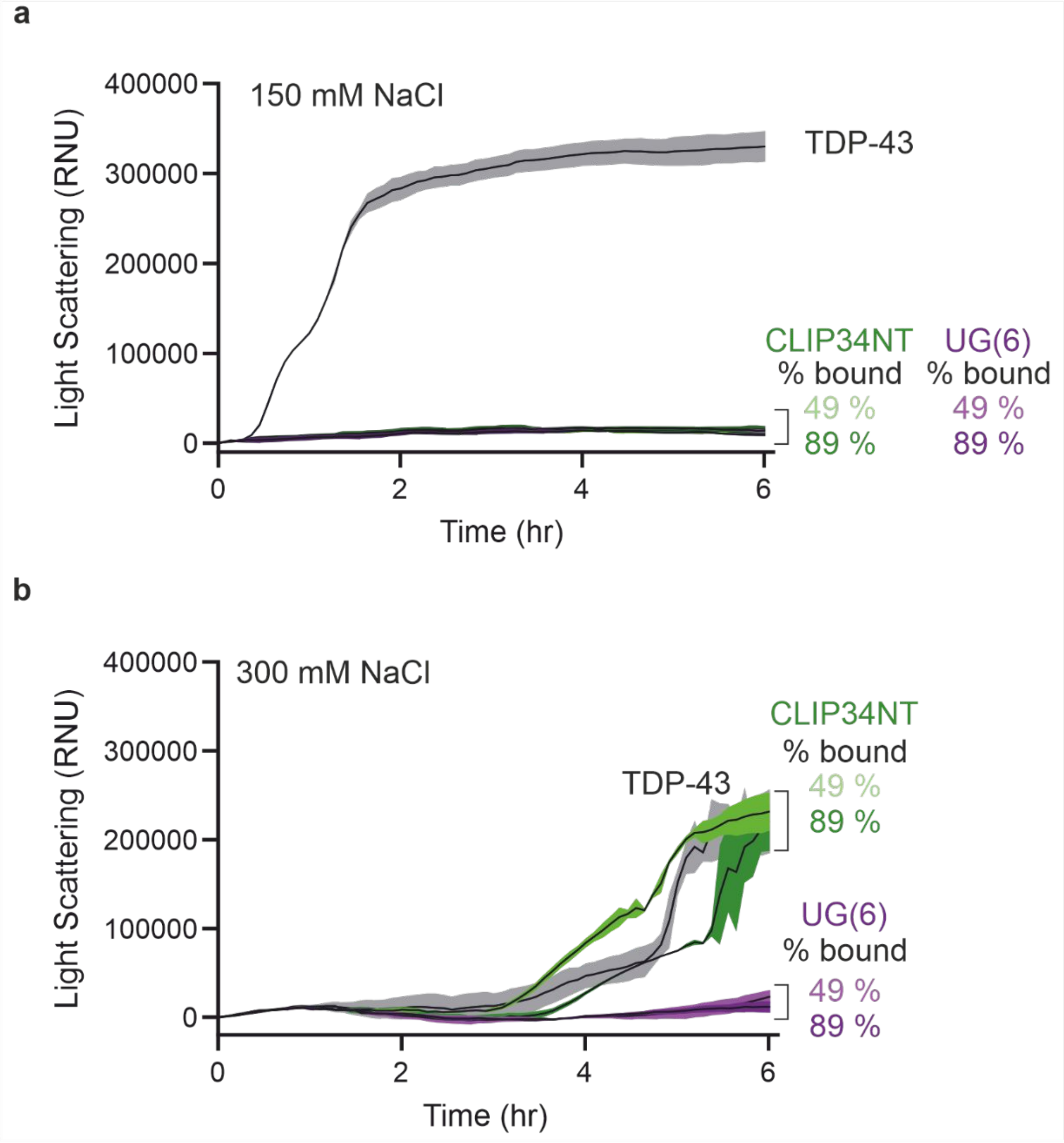

Supplementary Figure 5. Salt and RNA sequence modulate RNA-mediated antagonization of TDP-43 aggregation. Aggregation of TDP-43 in the presence of CLIP34NT and UG(6) in **(a)** 150 mM NaCl-containing buffer or **(b)** 300 mM NaCl-containing buffer. Two concentrations of RNA were tested corresponding to 49 or 89 % bound TDP-43. RNU = relative nephelometry units. The mean of three independent experiments is shown, and the shaded regions show the standard deviation of the measurements. Note that the TDP-43 construct use contains a C-terminal MBP tag for solubility and the aggregation reaction is triggered by addition of TEV protease. See **Supplementary Table 2** for RNA concentrations used to achieve the different % bound states.

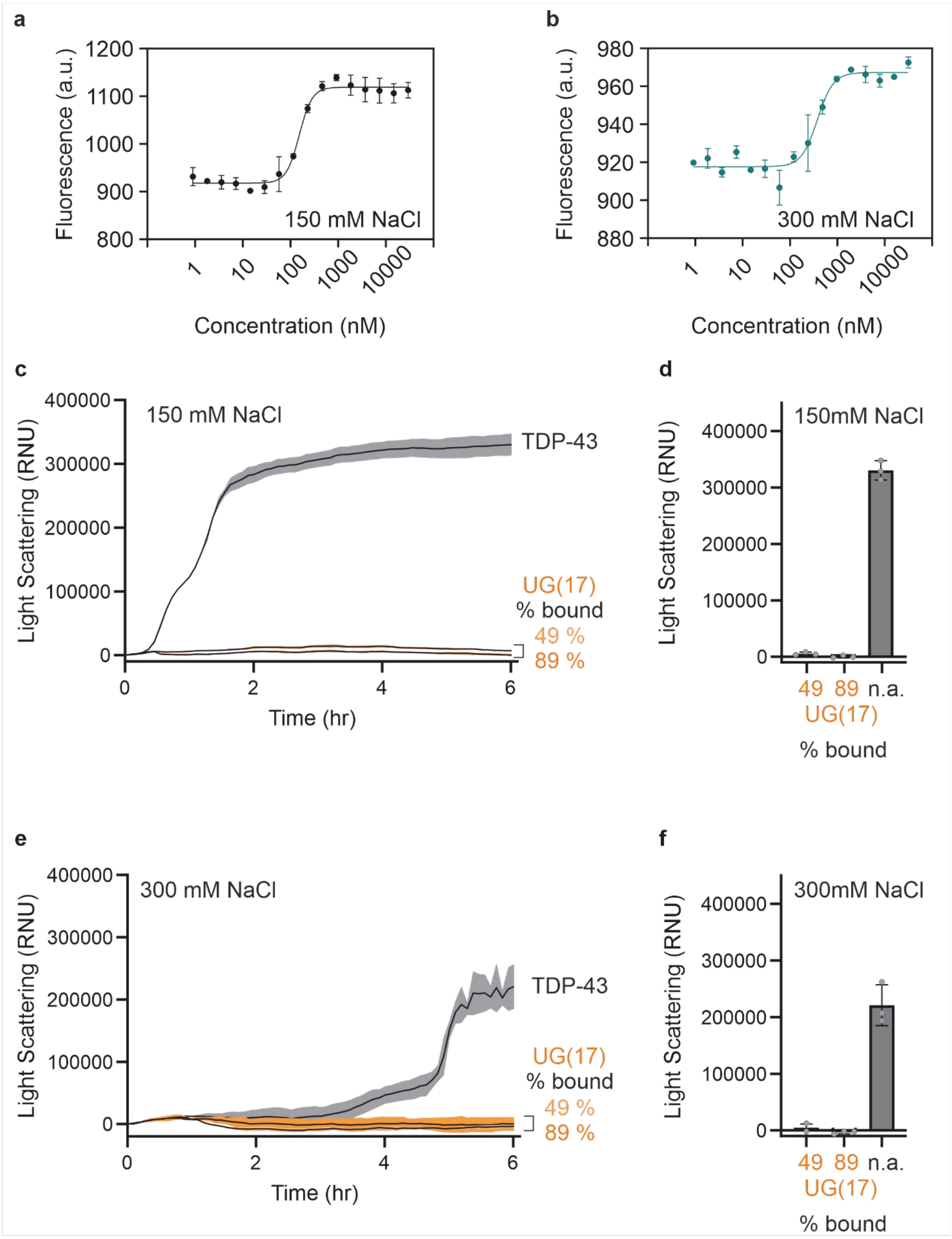

Supplementary Figure 6. A longer UG-rich oligonucleotide, UG(17), behaves in the same fashion as UG(6). MST binding curves for TDP-43 binding to UG(17) in **(a)** 150 mM and **(b)** 300 mM NaCl containing buffers. Two replicates were performed and averaged prior to fitting, and the

error (standard deviation) between replicates is plotted. See methods for experimental details and details of fitting procedure. See **Supplementary Table 2** for fitted EC_{50} and Hill coefficient values. **(c-f)** Aggregation of TDP-43 in the absence/presence of UG(17) in **(c,d)** 150 mM NaCl-containing buffer or **(e,f)** 300 mM NaCl-containing buffer. Two concentrations of RNA were tested corresponding to 49 or 89 % bound TDP-43. RNU = relative nephelometry units. **(c and e)** show the aggregation kinetics, and the mean of three independent experiments is shown, while the shaded regions show the standard deviation of the measurements. Note that the TDP-43 construct use contains a C-terminal MBP tag for solubility and the aggregation reaction is triggered by addition of TEV protease. **(d and f)** show the light scattering measurements after 6 hr incubation from **c and e**. Bars indicate the mean of three independent experiments, and the error bar shows the standard deviation. Individual data points are shown. See **Supplementary Table 2** for RNA concentrations used to achieve the different % bound states.

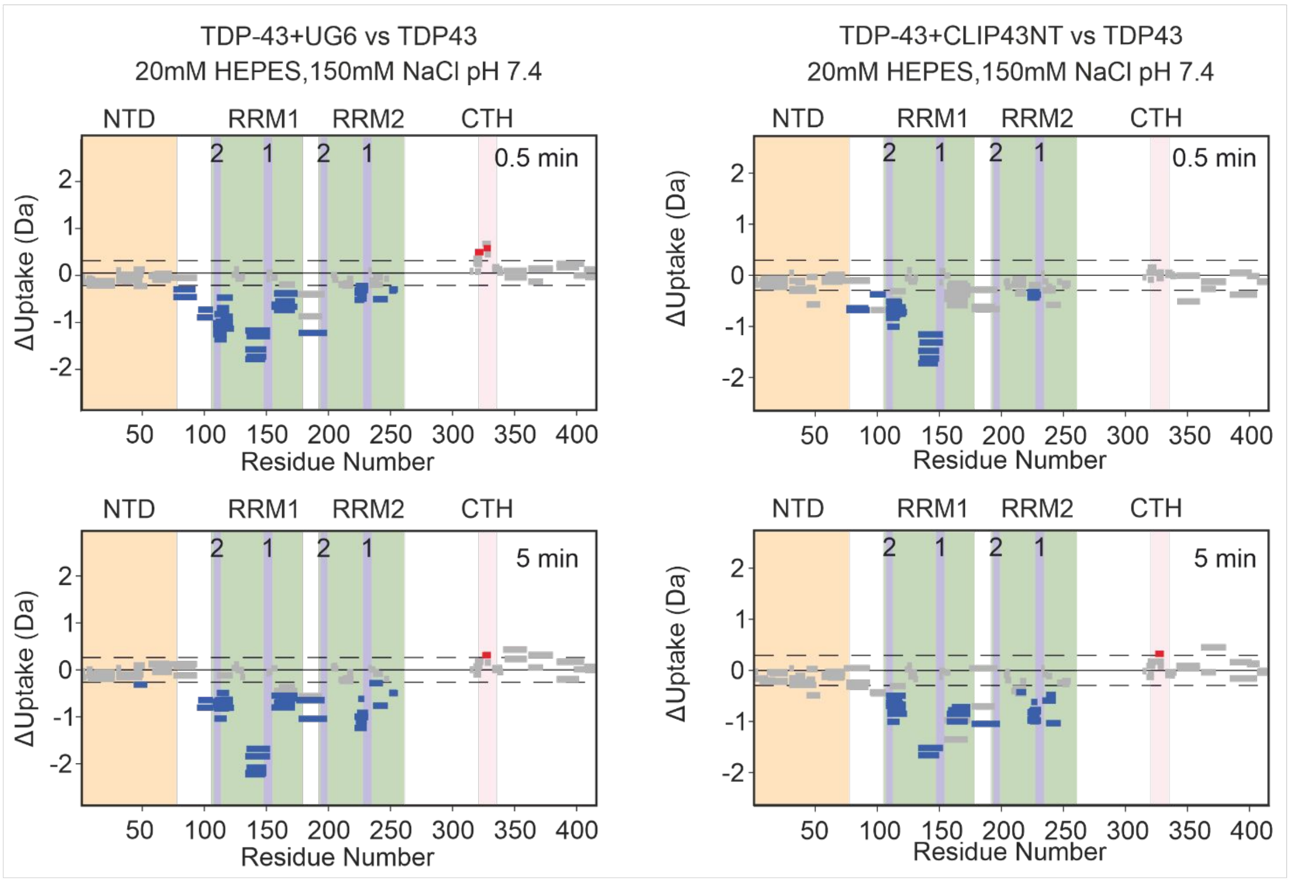

Supplementary Figure 7. Wood's plots showing the difference in deuterium uptake in TDP-43 in 150 mM NaCl-containing buffer at a 0.5 and 5 min HDX timepoint, comparing TDP-43 alone with TDP-43 in the presence of UG(6) (left) or CLIP34NT (right). Wood's plots were generated using Deuteros 2.0. Peptides coloured in blue or red, respectively, are protected or deprotected from exchange in the presence of CLIP43NT/UG(6). The NTD (orange), RRMs (green) and CTH (pink) are indicated as shaded regions. RNP-1 and RNP-2 motifs in RRM1 and RRM2 are labelled and indicated by purple shading. Peptides with no significant difference between conditions, determined using a hybrid significance test[1] with a 98 % confidence interval are shown in grey. Note that the hybrid significance test comprises two components: a t-test at the peptide level and an estimated global significance cutoff based on an estimation of the experimental error (see ref.[1]), indicated here by the dotted line. To meet the criteria for significance, each peptide must pass both tests, and therefore some peptides which lie outside the global significance cutoffs are not statistically significantly different. Such a strategy has been reported to reduce the risk of false positives[1]. See Methods for experimental details. The Wood's plot for the 2 min timepoint can be found in **Figure 4.**

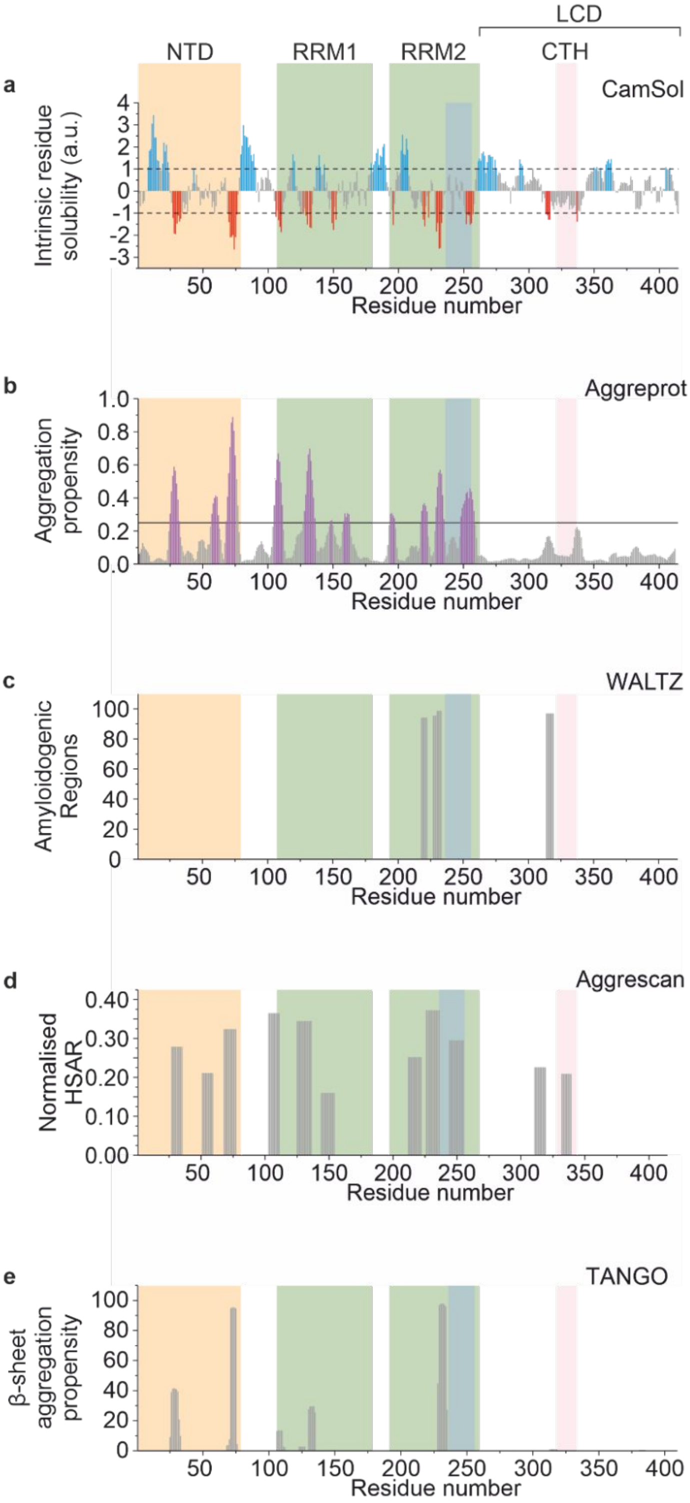

Supplementary Figure 8: Analysis of TDP-43 using aggregation prediction algorithms. (a) Camsol[2,3], **(b)** Aggreprot[4], **(c)** WALTZ[5], **(d)** Aggrescan[6], and **(d)** TANGO[7–9] profiles of TDP-43. In **(a)** residues below the threshold bar (-1, dashed line) indicated as red bars are aggregation promoting, residues above the threshold bar (1, dashed line) indicated as blue bars are solubility promoting. In **(b)** purple bars indicate aggregation prone residues which have an Aggreprot score above the threshold value of 0.25[4]. In **(c-e)** grey bars indicate regions that are predicted to be aggregation prone by the algorithms used. HSAR = hot spot area per residue. Orange shading indicates the NTD, RRMs are indicated with green shading, CTH is indicated with red shading. The blue shaded region corresponds to the sequence ^{247}DLIIKGISVHI^{257}. This region is highlighted by the Camsol, Aggreprot, Aggrescan, and Zyggregator (in[10]) algorithms.

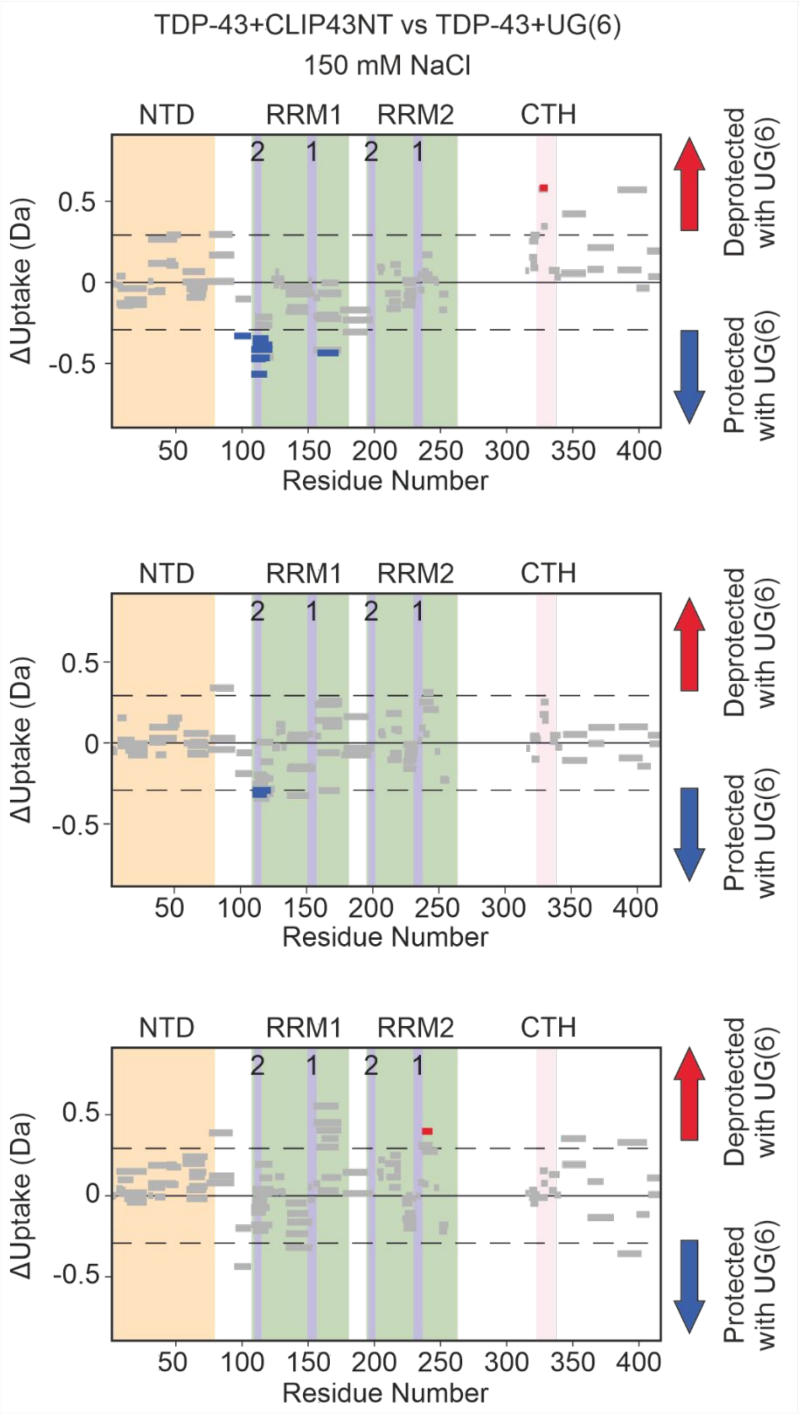

Supplementary Figure 9: Wood's plots showing the difference in deuterium uptake in TDP-43 in 150 mM NaCl-containing buffer at 0.5 (top), 2 (middle) and 5 (bottom) min timepoints, comparing TDP-43 bound to CLIP43NT and TDP-43 bound to UG(6). Wood's plots were generated using Deuteros 2.0. Peptides coloured in blue are protected from exchange in the presence of UG(6) relative to CLIP34NT. The NTD (orange), RRMs (green) and CTH (pink) are indicated as shaded regions. RNP-1 and RNP-2 motifs in RRM1 and RRM2 are labelled and indicated by purple shading. Peptides with no significant difference between conditions, determined using a hybrid significance test[1] with a 98 % confidence interval are shown in grey. Note that the hybrid significance test comprises two components: a t-test at the peptide level and an estimated global significance cutoff based on an estimation of the experimental error (see ref.[1]), indicated here by the dotted line. To meet the criteria for significance, each peptide must pass both tests, and therefore some peptides which lie outside the global significance cutoffs are not statistically significantly different. Such a strategy has been reported to reduce the risk of false positives[1].

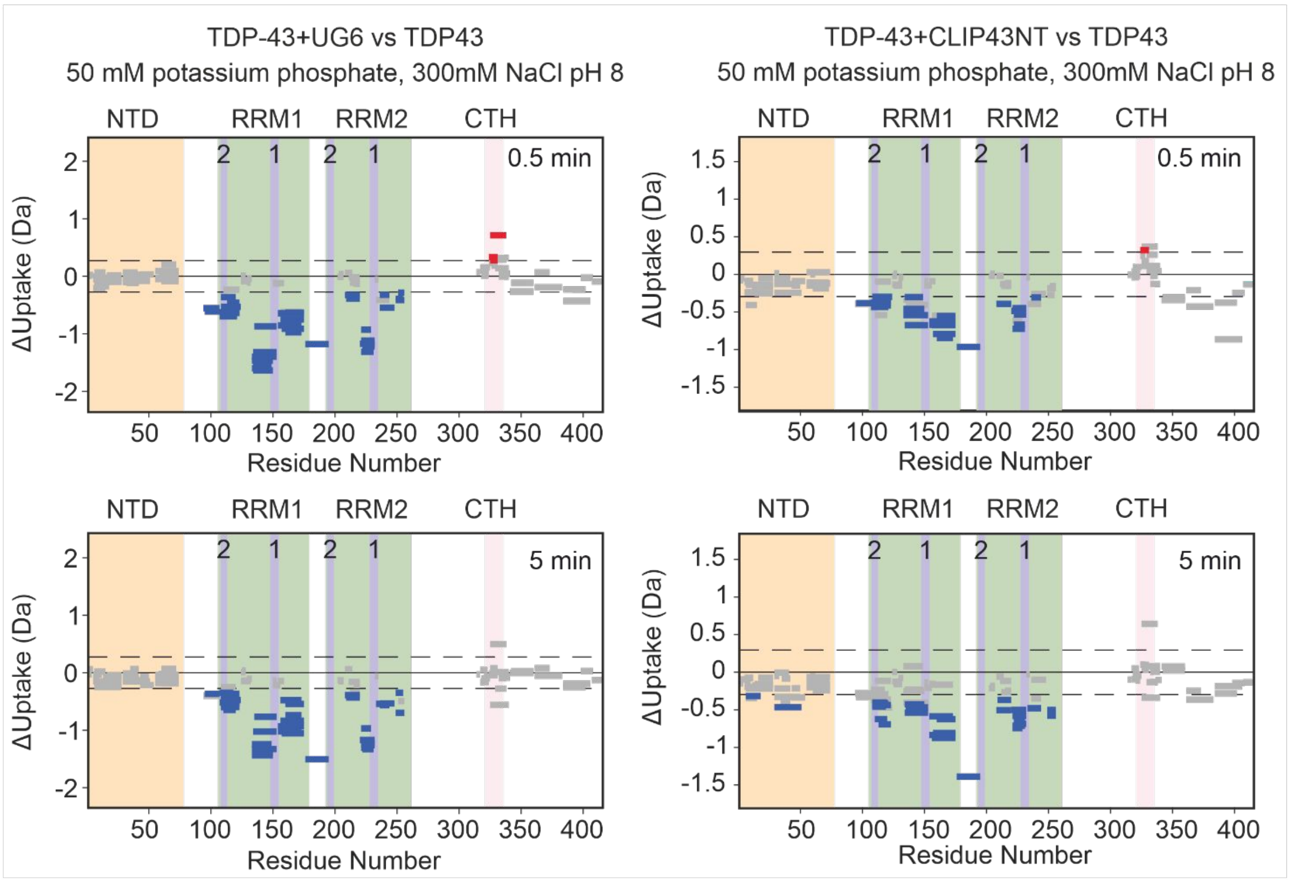

Supplementary Figure 10: Wood's plots showing the difference in deuterium uptake in TDP-43 in 300 mM mM NaCl-containing buffer at a 0.5 and 5 min HDX timepoint, comparing TDP-43 alone with TDP-43 in the presence of UG(6) (left) or CLIP34NT (right). Wood's plots were generated using Deuteros 2.0. Peptides coloured in blue or red, respectively, are protected or deprotected from exchange in the presence of CLIP43NT/UG(6). The NTD (orange), RRMs (green) and CTH (pink) are indicated as shaded regions. RNP-1 and RNP-2 motifs in RRM1 and RRM2 are labelled and indicated by purple shading. Peptides with no significant difference between conditions, determined using a hybrid significance test[1] with a 98 % confidence interval are shown in grey. Note that the hybrid significance test comprises two components: a t-test at the peptide level and an estimated global significance cutoff based on an estimation of the experimental error (see ref.[1]), indicated here by the dotted line. To meet the criteria for significance, each peptide must pass both tests, and therefore some peptides which lie outside the global significance cutoffs are not statistically significantly different. Such a strategy has been reported to reduce the risk of false positives[1]. See Methods for experimental details. The Wood's plot for the 2 min timepoint can be found in **Figure 5.**

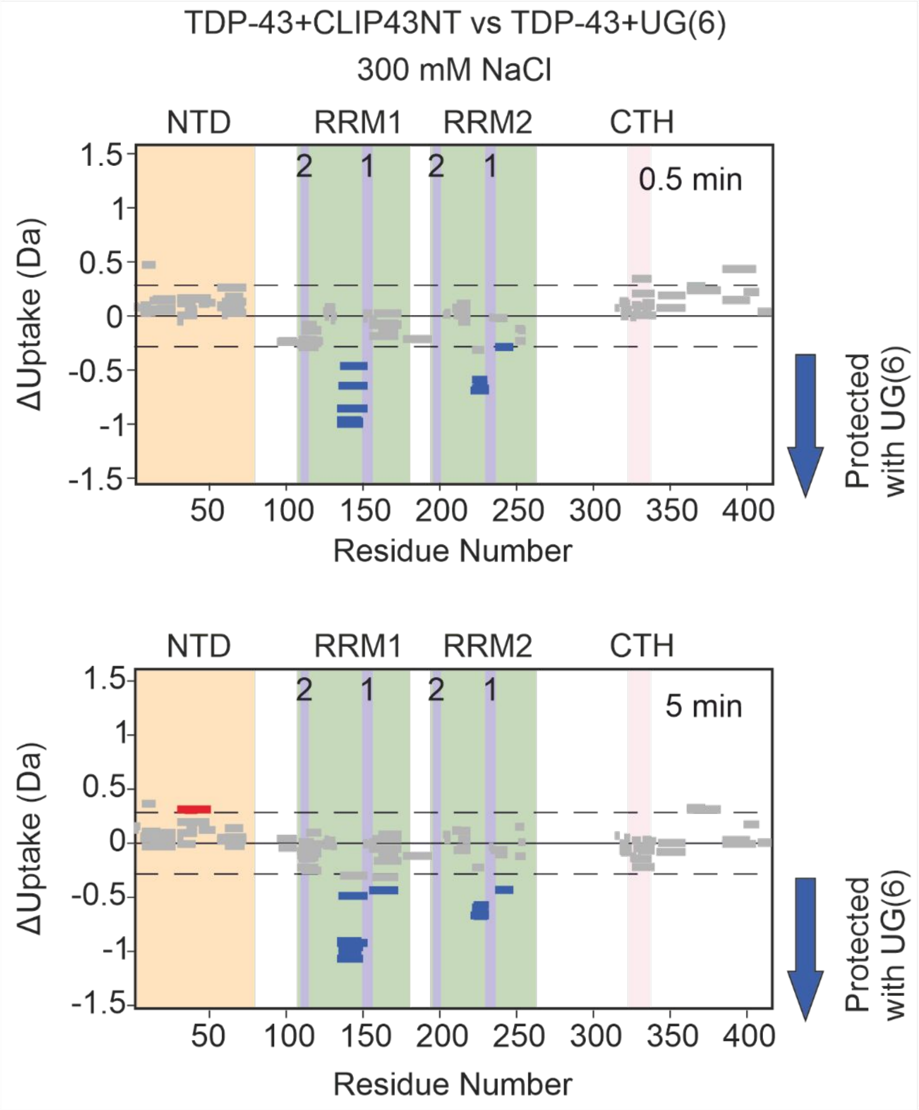

Supplementary Figure 11: Wood's plots showing the difference in deuterium uptake in TDP-43 in 300 mM NaCl-containing buffer at 0.5 and 5 (bottom) min timepoints, comparing TDP-43 bound to CLIP43NT and TDP-43 bound to UG(6). Wood's plots were generated using Deuteros 2.0. Peptides coloured in blue are protected from exchange in the presence of UG(6) relative to CLIP34NT. The NTD (orange), RRMs (green) and CTH (pink) are indicated as shaded regions. RNP-1 and RNP-2 motifs in RRM1 and RRM2 are labelled and indicated by purple shading. Peptides with no significant difference between conditions, determined using a hybrid significance test[1] with a 98 % confidence interval are shown in grey. Note that the hybrid significance test comprises two components: a t-test at the peptide level and an estimated global significance cutoff based on an estimation of the experimental error (see ref.[1]), indicated here by the dotted line. To meet the criteria for significance, each peptide must pass both tests, and therefore some peptides which lie outside the global significance cutoffs are not statistically significantly different. Such a strategy has been reported to reduce the risk of false positives[1]. The Wood's plot for the 2 min timepoint can be found in **Figure 6.**

Supplementary Table 1. Binding affinities of RNA oligonucleotides to TDP-43 measured by MST. Each interaction was measured in duplicate and data points averaged prior to fitting (see Methods). Coeff = coefficient.

	**CLIP34NT**		**UG(6)**		**UG(17)**	
	**EC50 (nM)**	**Hill Coeff.**	**EC50 (nM)**	**Hill Coeff.**	**EC50 (nM)**	**Hill Coeff.**
**20mM HEPES,150mM NaCl, pH 7.4**	384	3.6	356	5.0	153	3.2
**50 mM potassium phosphate, 300mM NaCl, pH 8**	1169	2.2	373	5.0	361	2.6

Supplementary Table 2. Concentration of each RNA oligonucleotide added to nephelometry experiments to achieve different degrees of TDP-43 saturation with RNA under different solution conditions (**Figure 3, main text, Supplementary Figures 5 and 6**). Affinities from **Supplementary Table 1** were used to calculate these concentrations. The TDP-43 concentration in each experiment was 10 μM.

		% Bound TDP-43	
Buffer	RNA oligonucleotide	49%	89%
20mM HEPES,150mM NaCl, pH 7.4	CLIP34NT	5.3 μM	12 μM
	UG(6)	5.3 μM	12 μM
	UG(17)	5 μM	10.2 μM
50 mM potassium phosphate, 300mM NaCl, pH 8	CLIP34NT	6 μM	18.5 μM
	UG(6)	5.3 μM	12 μM
	UG(17)	5.3 μM	12 μM

Supplementary Table 3: HDX-MS Data Summary Table. SD = standard deviation, CI = confidence interval.

Data Set	TDP-43	TDP-43 + CLIP34NT	TDP-43 + UG(6)	TDP-43	TDP-43 + CLIP34NT	TDP-43 + UG(6)
HDX reaction details	20 mM HEPES, 150 mM NaCl, pD 7.4, 4 ºC			50 mM potassium phosphate, 300mM NaCl pD 8, 4 ºC		
HDX time course (min)	0.5, 2, 5 min			0.5, 2, 5 min		
HDX control samples	t = 24 hr in 8 M d_4-urea at 4ºC			t = 24 hr in 8 M d_4-urea at 4ºC		
Back-exchange (mean/interquartile range)	46.8% / 17.3%			56.4% / 18.1%		
# of Peptides	114	114	114	116	116	116
Sequence coverage	85.34%	85.34%	85.34%	80.29%	80.29%	80.29%
Average peptide length / Redundancy	12.41 / 3.99	12.41 / 3.99	12.41 / 3.99	11.80 / 4.10	11.80 / 4.10	11.80 / 4.10
Replicates	3 (technical)	3 (technical)	3 (technical)	3 (technical)	3 (technical)	3 (technical)
Repeatability (average SD)	0.061	0.07	0.058	0.067	0.067	0.057
Significant differences in HDX (delta HDX > X D)	Reference	Hybrid Significance test: 98% CI: 0.29 Da / p-value <0.02	Hybrid Significance test: 98% CI: 0.26 Da / p-value <0.02	Reference	Hybrid Significance test: 98% CI: 0.30 Da / p-value <0.02	Hybrid Significance test: 98% CI: 0.27 Da / p-value <0.02

Supplementary References

[1] T. S. Hageman, D. D. Weis, *Anal. Chem.* **2019**, *91*, 8008–8016.
[2] P. Sormanni, F. A. Aprile, M. Vendruscolo, *J. Mol. Biol.* **2015**, *427*, 478–490.
[3] P. Sormanni, L. Amery, S. Ekizoglou, M. Vendruscolo, B. Popovic, *Sci. Rep.* **2017**, *7*, 8200.
[4] J. Planas-Iglesias, S. Borko, J. Swiatkowski, M. Elias, M. Havlasek, O. Salamon, E. Grakova, A. Kunka, T. Martinovic, J. Damborsky, J. Martinovic, D. Bednar, *Nucleic Acids Res.* **2024**, *52*, W159–W169.
[5] J. Beerten, J. Van Durme, R. Gallardo, E. Capriotti, L. Serpell, F. Rousseau, J. Schymkowitz, *Bioinformatics* **2015**, *31*, 1698–1700.
[6] O. Conchillo-Solé, N. S. de Groot, F. X. Avilés, J. Vendrell, X. Daura, S. Ventura, *BMC Bioinformatics* **2007**, *8*, 65.
[7] A.-M. Fernandez-Escamilla, F. Rousseau, J. Schymkowitz, L. Serrano, *Nat. Biotechnol.* **2004**, *22*, 1302–1306.
[8] R. Linding, J. Schymkowitz, F. Rousseau, F. Diella, L. Serrano, *J. Mol. Biol.* **2004**, *342*, 345–353.
[9] F. Rousseau, J. Schymkowitz, L. Serrano, *Curr. Opin. Struct. Biol.* **2006**, *16*, 118–126.
[10] E. L. Guenther, P. Ge, H. Trinh, M. R. Sawaya, D. Cascio, D. R. Boyer, T. Gonen, Z. H. Zhou, D. S. Eisenberg, *Nat. Struct. Mol. Biol.* **2018**, *25*, 311–319.
